# Aldehyde Electrophilicity and Ring Strain Govern Xylose Acetalization Pathways for Biobased Chemical Production

**DOI:** 10.1002/cssc.202501562

**Published:** 2025-12-29

**Authors:** Zezhong John Li, Deep M. Patel, Songlan Sun, Claire L. Bourmaud, Tso‐Hsuan Chen, Dionisios G. Vlachos, Jeremy S. Luterbacher

**Affiliations:** ^1^ Laboratory of Sustainable and Catalytic Processing (LPDC) Institute of Chemicals Sciences and Engineering (ISIC) School of Basic Sciences (SB) École Polytechnique Fédérale de Lausanne Lausanne Switzerland; ^2^ Center for Catalytic Science and Technology and Delaware Energy Institute Department of Chemical and Biomolecular Engineering University of Delaware Newark Delaware USA

**Keywords:** acetalization, carbohydrates, electrophilicity, reaction mechanisms, ring strain, xylose

## Abstract

Xylose acetalization has emerged as a potent tool to extract this sugar from lignocellulosic biomass and for creating new biobased chemicals and materials. This article elucidates a generalized reaction network for xylose acetalization and reveals the role of aldehyde electrophilicity and ring strain in intermediate formation. Aldehydes with strong electrophilicity stabilize xylose as both furanose‐ and pyranose‐monoacetals, whereas weaker aldehydes favour xylofuranose acetalization due to the high ring strain in pyranose acetals. The energetically favoured furanose diacetals dominate the product distribution over extended reaction time regardless of aldehyde types and reaction pathways. Measurements of the xylose tautomer ratio in the reaction conditions highlighted the importance of xylose isomerization in forming furanose acetals. These mechanistic insights not only explain the evolution of reaction intermediates but also aid in identifying potential products for sustainable chemical synthesis.

## Graphical abstract

Reaction kinetics and operando heteronuclear single quantum coherence nuclear magnetic resonance studies revealed the reaction pathway of xylose acetalization with different aldehydes. Product distribution and yields were significantly influenced by aldehyde electrophilicity and the orientation of xylose hydroxyl groups.

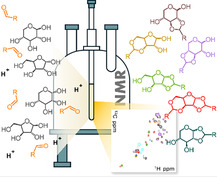

## 1 Introduction

In the pursuit of sustainable and environmentally friendly chemical production, there is a pressing need to transition from fossil‐based feedstocks to renewable alternatives. Biomass‐derived carbohydrates have emerged as a particularly promising feedstock due to their abundance in the natural world and potential carbon‐neutral production. A vast body of literature has reported numerous approaches to converting sugars into platform chemicals, for instance, furfural, xylitol, levulinic acid, and 5‐hydroxymethylfurfural via dehydration, hydrogenation, or a successive combination of both [1, 2]. However, minimizing the modification of the sugar molecule during conversion would better conform to the principles of green chemistry, as excessive structural alterations often introduce processing complexity, energy‐intensive steps, and undesirable byproducts. One strategy is to directly introduce functional groups to the native sugar structure, leveraging the abundant hydroxyl groups in carbohydrates. Notably, our group [3, 4] and others [5, 6, 7, 8] have reported reacting d‐xylose, which is the second most abundant sugar on the planet after glucose, with various aldehydes to form cyclic acetals between contiguous hydroxyl groups in the sugar molecule. These one‐step reactions directly converted the aldopentose into a polar aprotic solvent (diformylxylose, DFX) [9], a diacid as a polymer building block (diglyoxylic acid xylose) [10], and a host of amphiphiles as surfactants [11]. Compared to glucose, the valorization of xylose does not compete with the food supply. These acetalization strategies could open up new avenues for xylose valorization, converting a refinery byproduct to value‐added chemicals and materials.

**FIGURE 1:**
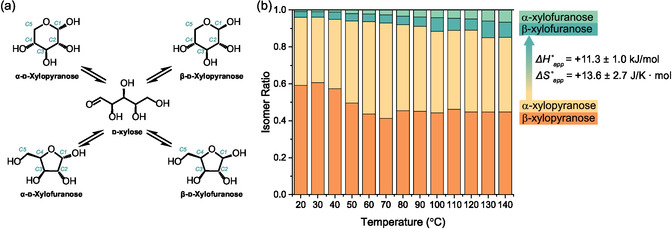
(a) Molecular structures of d‐xylose tautomers in Haworth projections [14]. The carbon atom numbering convention is shown in cyan. Nonpolar hydrogens are omitted for clarity. After acetalization, the numbering remains unchanged in this work. (b) Isomer ratios of d‐xylose tautomers in 1,4‐dioxane with 5 vol.% water as a function of temperature measured using operando ^1^H NMR. The reaction enthalpy and entropy were calculated from the measured equilibrium constants (Table S3).

Although the intrinsic mechanism of acetalization between aldehydes and alcohols has been widely understood [12, 13], the acetalization of monosaccharides is complicated by their tautomerization in solutions. Monosaccharides undergo thermodynamically controlled ring‐chain isomerization, resulting in an anomeric equilibrium of five isomers (the example of d‐xylose is shown in Figure 1a) [14]. The equilibrium ratio of these isomers heavily depends on the solvent and the temperature [15, 16], but we are not aware of any reports of the xylose isomer ratio in conditions above room temperature in nonaqueous solutions (i.e. conditions that have been very relevant to xylose conversion in recent years) [17, 18, 19].

The tautomer structure is especially relevant because the steric effect of hydroxyl groups in monosaccharides (i.e., the axial and equatorial positions on a furanose or pyranose ring) imposes conformational constraints on the possible acetalization pathways and products. Existing studies on monosaccharide acetalization revealed that the reactions yielded a dioxane or dioxolane ring fused to a furanose or pyranose ring [20, 21, 22]. Such structures can be particularly susceptible to the initial ‐OH conformations compared to similar reactions between aldehydes and mono‐ols or linear polyols, which may explain why the reaction selectivity and yield can strongly vary by the sugar type [23]. Since the aldehydes in the available literature were introduced mainly as protection groups for hydroxyl functionalities, they were often limited to a few well‐known species (e.g., acetone and benzaldehyde). Also, the formed acetals were unstable by design to enable subsequent deprotection. Such protection strategies were used for many sugars, including xylose [24]. However, the primary focus was always improving the yield of the fully protected aldopentose instead of identifying intermediates and reaction pathways [25].

In the context of direct saccharide functionalization via acetalization, various intermediates should be identified, as they could become additional or alternative products of interest. Systematic aldehyde testing could reveal the effect of aldehyde types on the yield and selectivity. In this work, we performed a mechanistic study of d‐xylose acetalization with various aldehydes by tracking reaction intermediates with operando nuclear magnetic resonance (NMR) spectroscopy. As a result, we propose a mechanism and reaction network which highlights the interplay of the aldehyde electrophilicity and ring strain of the resulting cyclic acetals. *Ab initio* calculations were conducted to quantify the strain on different ring conformations. Overall, this study provides a detailed map of xylose acetalization that can be used to understand and improve the selectivity and yield of various acetalization products, which ultimately guides product design and methodology development.

## 2 Results and Discussion

Given the importance of the initial xylose tautomer structure on subsequent valorization pathways and the lack of understanding of these structures in the relevant conditions, we measured the equilibrium tautomer ratios of xylose between 20 and 140°C in 1,4‐dioxane with 5% water using quantitative ^1^H‐NMR (Figure 1b, additional discussions in Section S7). The apparent standard reaction enthalpy and entropy were calculated as 11.3 ± 1.0 kJ/mol and 13.6 ± 2.7 J/(K mol), respectively, suggesting that the conversion from xylopyranose to xylofuranose is more entropically driven, consistent with what is known for d‐glucose isomerization [26]. The resulting tautomer ratios measured here, as well as these thermodynamic parameters, can be used to understand subsequent acetalization reactions and potentially inform future xylose valorization studies.

**FIGURE 2:**
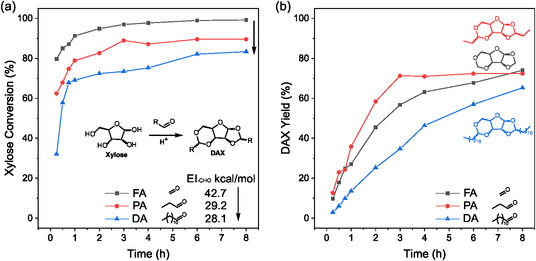
(a) Xylose conversions and (b) DAX yields over the reaction course when xylose reacted with formaldehyde (FA), propionaldehyde (PA), and dodecanal (DA), respectively. Experiments were performed at 60°C in 1,4‐dioxane with a 2:1 aldehyde to xylose molar ratio and 1.6 vol.% H_2_SO_4_. EI stands for the electrophilicity index. The xylose conversion was measured by high‐performance liquid chromatography (HPLC), and the diacetal yield by gas chromatography (GC).

The acetalization reaction between d‐xylose and formaldehyde was first reported by de Bruyn and van Ekenstein in 1903 to form a readily crystallizable product, whose structure was determined as 1, 2; 3, 5‐O‐dimethylene‐d‐*α*‐xylofuranose (or diformylxylose, DFX in short) by Schmidt and Nieswandt in 1949 [27, 28]. When expanding the reaction scope to more aldehydes, we denote the general structure of such d‐xylose diacetals as 1, 2; 3, 5‐*O*‐dialkylene‐d‐xylofuranose (DAX, see structure in Figure 2). Formaldehyde (FA), propionaldehyde (PA), and dodecanal (DA) were first used to illustrate the effect of aldehyde functionality on acetalization kinetics, using H_2_SO_4_ as a homogenous Brønsted acid catalyst. While the intrinsic acetalization mechanism between an aldehyde and an alcohol catalyzed by a homogeneous acid is well understood [12, 13], we performed a detailed reaction pathway analysis and elucidated the mechanism specifically for xylose acetalization (vide infra). The rate of xylose consumption increased with decreasing aldehyde side chain length (rate: FA > PA > DA, Figure 2a). However, the rate of DAX formation did not follow the same order (Figure 2b). For instance, while FA resulted in a faster sugar consumption compared to PA, it led to a slower DAX formation. The significant gap between the sugar conversion and the DAX yield in all cases, especially at the beginning of the reaction, strongly indicated the formation of intermediates. This naturally raised the question of what intermediates were formed and how the type of aldehydes affected their yields.

### 2.1 The Effect of Aldehyde Electrophilicity

**FIGURE 3:**
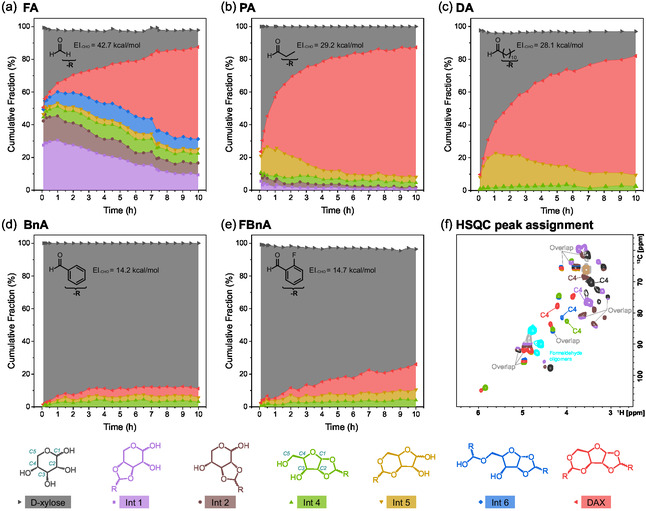
The molar fraction of xylose, intermediates, and products as a function of reaction time when reacting with (a) formaldehyde (FA), (b) propionaldehyde (PA), (c) dodecanal (DA), (d) benzaldehyde (BnA), and (e) 2‐fluorobenzaldehyde (FBnA), measured by operando NMR. Reactions were conducted at 60°C in dioxane‐d8 with a 2:1 aldehyde to xylose molar ratio and 1.6 vol.% D_2_SO_4_. One pyranose structure and one furanose structure are numbered in cyan as examples for easy reference. The aldehyde used and the corresponding ‐R groups in these molecules are labelled in each panel. (f) Identification of ^1^H‐^13^C HSQC cross peaks of intermediates and products in operando NMR in the reaction of xylose and formaldehyde. The cross peaks corresponding to the C4 of xylose and its subsequent intermediates were used to measure relative yields. Example ^1^H‐^13^C HSQC spectra of xylose reacting with other aldehydes are presented in Figures S6–S9.

Due to the large number of possible intermediates with structures similar to the product and the occasional instability of these intermediates even outside of reaction conditions, their separation through various chromatographic techniques was very challenging. Instead, we were able to use operando NMR to capture the evolution of intermediates throughout the reaction course (Figure 3, with the method detailed in Supporting Information Section S2). Briefly, small quantities of intermediates were separated using a high‐performance liquid chromatography (HPLC) fraction collector. Their structures were identified using a series of NMR sequences and mass spectroscopy (Section S3). Reactions were carried out inside the NMR spectrometer, where ^1^H‐^13^C heteronuclear single quantum coherence (HSQC) spectra were collected every 6 min. In each spectrum, peak volumes were integrated and correlated to the quantities of each identified compound (Section S5). HSQC spectra were used because ^1^H spectra were too crowded to be interpreted, even though they could be theoretically quantitative. While HSQC integration is not strictly quantitative, it can be used as a comparative tool to approximate reaction kinetics, assuming little variations of relaxation rate amongst analyzed molecules. This assumption was further validated by measuring the T_2_ relaxation constant of various intermediates (Section S6). The same method was also previously applied and validated in biomass fractionation [29]. Note that 1,4‐dioxane‐d8 was selected as the solvent, as greener alternatives were not commercially available in deuterated forms. Nonetheless, the green solvents, such as 2‐methyl‐THF, can be used as a good alternative for synthesis [9]. Reaction rate trends (i.e. fastest for FA, followed by PA and then DA) were consistent for quantitative HPLC/gas chromatography data (Figure 2) and Operando HSQC NMR data (Figure 3). The deviation in the exact yields from Figure 1 might have stemmed from the use of a deuterated solvent and a lack of stirring in the spectrometer. Among the intermediates, **Int 1** and **2** are monoacetals formed between the contiguous hydroxyl groups in d‐xylopyranose (3,4‐*O*‐ and 2,3‐*O*‐acetals, respectively), which were particularly prevalent in the reaction with formaldehyde. **Int 4** and **5** are monoacetals formed between the contiguous hydroxyl groups in d‐xylofuranose (1,2‐*O*‐ and 3,5‐*O*‐acetals, respectively), detected in reactions with all aldehydes. **Int 6** had the same structure as **Int 4** with an additional hemiacetal between an aldehyde and the C5–OH, which was only observed in the reaction with formaldehyde.

The reaction between formaldehyde (FA) and d‐xylose led to a distinctively different product distribution compared to other aldehydes (Figure 3a): (i) large quantities of pyranose‐based intermediates (**Int 1** and **2**) was formed despite that the final product DAX was furanose‐based; (ii) **Int 5** was formed at a much lower yield than **Int 4** and **6** throughout the reaction. Dodecanal (DA) produced more **Int 5** than propionaldehyde (PA) (Figure 3b,c). These differences likely arose from the different reactivity of aldehydes. The electrophilicity of the carbonyl carbon depends on the type and the number of its substituents [30]. In the presence of an alkyl substituent, the positive charge on the carbon atom can be better distributed due to alkyl chain positive inductive (+I) effect, leading to a lower charge density, and hence, lower electrophilicity and lower reactivity with hydroxyl groups in xylose [31]. The strength of the +I effect decreases with the alkyl chain size and the number of constituents on the adjacent carbon [31]. To quantify these differences, we calculated the electrophilicity indices of ‐CHO groups of various aldehydes (EI_‐CHO_ in Table S4) using the method proposed by Parr et al., employing *ab initio* calculations at the M06‐2X theory level with the def2‐TZVP basis set [32]. The EI_‐CHO_ decreases consistently from formaldehyde (FA) to dodecanal (DA). Additionally, the aromatic aldehydes have a lower EI_‐CHO_ than the saturated aldehydes. Overall, the density functional theory (DFT)‐calculated EI_‐CHO_ correlates with the sugar conversion rates (Figure 2a). The equilibrium selectivity of pyranose monoacetals is also positively correlated with the aldehyde's EI_‐CHO_, with a threshold of 28–29 kcal/mol, below which no pyranose acetals were observed (Figure S18). The same experiment was repeated using benzaldehyde and 2‐fluorobenzaldehyde to verify the contribution of aldehyde electrophilicity. The positive charge on the carbonyl carbon was well distributed around the aromatic ring in benzaldehyde owing to its conjugated carbonyl and aromatic ring. As expected, the low electrophilicity of benzaldehyde carbonyl led to a fractional xylose conversion and DAX yield (Figure 3d). By substituting an ortho‐position hydrogen with an electron‐withdrawing fluorine group, both xylose conversion and DAX yield increased substantially (Figure 3e), which reaffirmed the relationship between the carbonyl electrophilicity and reaction rates.

We observed a substantially different distribution of intermediates during acetal formation with formaldehyde. In particular, substantial quantities of pyranose monoacetals **Int 1** (up to 30%) and **2** (up to 15%) were formed with formaldehyde, while these two intermediates were not (or barely) observed with other aldehydes. The stronger electrophilicity of the carbon in formaldehyde likely facilitated acetalization of xylopyranose, whose vicinal hydroxyls were predominantly in trans configurations (except for the C1‐C2 diols of the *α*‐anomer, see Figure 1a). Such trans‐configured diols generated greater angular and torsional strains when forming cyclic acetals, which might be compensated by the high electrophilicity of formaldehyde. However, aldehydes with a less electrophilic carbonyl were unable to overcome this energy barrier. With a lower EI than FA, PA use led to the formation of much smaller quantities of **Int 1** and **Int 2** (less than 5%), while the use of DA, BnA, and FBnA resulted in no detectable pyranose‐based intermediates. **Int 1** and **2** were formed with two contiguous hydroxyl groups in the trans‐position, which might cause a higher strain on the dioxolane ring that was unfavoured when less electrophilic aldehydes were used. The precise quantification of the ring energies of these intermediates is discussed in more detail in a later section.

### 2.2 Identifying a Reaction Pathway

**FIGURE 4:**
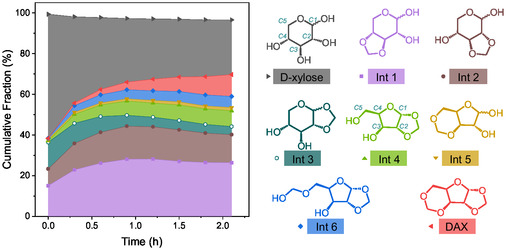
The molar fraction of xylose, intermediates, and products as a function of reaction time when reacting with formaldehyde, measured by operando HSQC after prior reaction under mild conditions. The aqueous reactant mixture of d‐xylose and formaldehyde‐d2 was initially left at 45°C for 30 min with 0.2 vol.% D_2_SO_4_ to reveal the formation of **Int 3** (ex situ). Then 25 µL of the resultant mixture was added to 0.5 mL of dioxane‐d8 with 1.6 vol.% D_2_SO_4_ and the mixture was heated to 60°C. Operando NMR measurements started (*t* =  0) once the temperature of the mixture reached 60°C.

If formaldehyde was sufficiently electrophilic to stabilize a dioxolane ring between trans‐positioned C3–OH and C4–OH, and between trans‐positioned C2–OH and C3–OH of xylopyranose (**Int 1** and **2**, respectively), a similar cyclic acetal should be able to form between C1–OH and C2–OH (i.e., xylopyranose 1,2‐*O*‐monoacetal or **Int 3**, Figure 4). However, such an intermediate was not initially isolated using an HPLC with a fraction collector or detected using the standard operando HSQC sequence at 60°C in dioxane‐d8 with 1.6 vol.% D_2_SO_4_ (the standard reaction conditions) (see Figure 3). Upon further investigation, the hypothesized **Int 3** was observed in operando with high‐resolution NMR spectroscopy under much milder conditions (45°C in D_2_O for 30 min with 0.2 vol.% D_2_SO_4_, see Figure S10). Specifically, both R and S stereocentres were detected at the C1 position of the **Int 3**. After diluting the aqueous mixture in 1,4‐dioxane‐d8, the **Int 3** fraction continuously decreased upon increasing the catalyst loading and the temperature to the standard reaction conditions (Figure 4). In comparison, **Int 1** and **2** followed the same trend as previously observed at all conditions: initial increase followed by gradual decay. The disappearance of **Int 3** at harsher conditions not only explains its difficult observation and isolation, but its limited presence compared to **Int 1** and **2** also suggests that an easier pathway for subsequent conversion of **Int 3** to DAX (in contrast to **Int 1** and **2**) may exist.

**FIGURE 5:**
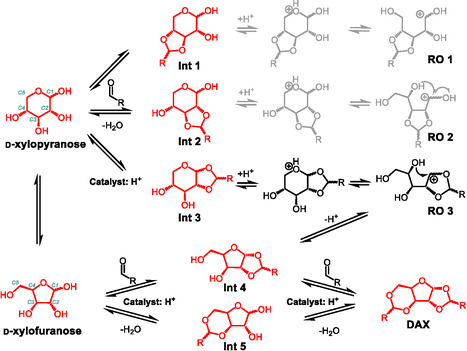
A proposed reaction network for d‐xylose acetalization with aldehydes. Molecules in red are experimentally observed. The pathways in gray do not proceed but are listed here as illustrations. One pyranose structure and one furanose structure are numbered in cyan as examples for easy reference. The ring‐opened intermediates are labelled as OR 1–3 corresponding to Int 1–3.

The different relative quantities of **Int 4** and **5**, with or without pyranose‐based intermediates (**Int 1–3**) provide a further suggestion for the reaction pathways. Formaldehyde resulted in a large fraction of pyranose monoacetals (Figures 3a and 4) and concurrently more **Int 4** than **Int 5**. In contrast, the use of propionaldehyde and dodecanal both led to negligible quantities of pyranose‐based intermediates and more **Int 5** than **Int 4**. The preferential formation of **Int 4** in the presence of pyranose‐based intermediates suggests a rationale for the steady decay of **Int 3** instead of **Int 1** and **2**: **Int 3** may have converted to **Int 4** through the reversible hydrolysis of the pyranose ring, similar to the well‐known sugar tautomerization mechanism (Figure 5) [33]. Briefly, the pyranose rings in **Int 1–3** could open through hydrolysis, forming the ring‐opened intermediates (**RO 1–3**), and the resulting carbocation could potentially form a furanose ring with the C4–OH. The already acetalized C4–OH in **RO 1** would inhibit this step. Though the C4–OH was free in **RO 2**, the dioxolane ring with trans‐positioned C2–O and C3–O likely posed a prohibitively high energy barrier to form a furanose ring, considering the two rings would be fused. In fact, furanose acetals were never observed with contiguous C–O bonds in trans‐conformation with any xylose‐aldehyde combinations [10, 34]. Therefore, **RO 1–2** could only reconstruct to the pyranose structures via the reverse reaction. Contrary to **RO 1** and **2**, the isomerization of **RO 3** to **Int 4** was not blocked by an occupied C4–OH, nor would the isomerization be completely prevented by a high energy barrier. As Ahsan et al. reviewed, the evolution of stereocentres can suggest the sequence of reaction steps during successive sugar dehydration and hydrogenation [35]. In HSQC, we observed that both R and S stereoisomers were present at the C1 position in **Int 3**. We propose that the C1 stereocentre could disappear through opening of the pyranose ring to form **RO 3** and then reconstruct either as a pyranose (back to **Int 3**) or to a furanose structure (**Int 4**), but the latter only with an R stereocentre at the C1 of **Int 4** (and subsequently DFX), which was what we observed experimentally. This phenomenon can be attributed to the aforementioned high energy barrier to form an S stereoisomer with the C1–O and C2–O in trans‐positions, while the R stereoisomer allows the C1–O and C2–O in cis‐positions. Combining the experimental measurements and theoretical assessments, we suggest that the pyranose‐based **Int 3** could undergo isomerization to form the furanose‐based **Int 4** (in addition to reverting back to xylose by deacetalization) (Figure 5).

Since we did not deduce any further transformations of **Int 1** and **2**, their eventual quantitative decay (as observed in Figure 3a) can be attributed to the reversible acetal hydrolysis that reforms xylose, which, in turn, is gradually consumed by either directly forming **Int 4** and **5** or forming **Int 3** that was continuously converted to **Int 4** (Figure 5). The steady formation in DAX from **Int 4** and **5** shifted the reaction equilibrium and ultimately consumed all intermediates. For most other aldehydes with weak carbonyl electrophilicity, we propose that xylose acetalization proceeds almost exclusively through furanose‐based intermediates (**Int 4** and **5**), despite the large portion of xylopyranose in the substrate under the reaction conditions (Figure 1b). The overwhelming presence of furanose acetal products, in contrast to the predominance of xylopyranose in the reactant mixture, suggests that continuous pyranose‐to‐furanose tautomerization is necessary to sustain acetal formation when the aldehyde is insufficiently electrophilic to stabilize pyranose acetals with trans‐positioned contiguous hydroxyl groups. In the case of a strong aldehyde like formaldehyde, the reaction notably proceeds through both the pyranose and furanose pathways, even though pyranose monoacetals are eventually converted to DAX from **Int 3** through **Int 4**, or from **Int 1** and **2** via reversible acetal hydrolysis. Similar impacts of vicinal hydroxyl orientations and aldehyde electrophilicity during the acetalization were observed for other monosaccharides, such as arabinose, lyxose, and glucose, which are discussed in Section S9. The following DFT calculations further support this interpretation, showing that the low‐energy pathways from xylose to DAX involve intermediate tautomerization, which minimizes ring strains and allows low energy barriers during the conversion.

In the recognition that industrial acetalization reactions will likely be carried out in greener solvents and may use solid‐acid catalysts, we have conducted an additional experiment to acetalize xylose with formaldehyde using HY zeolite as a heterogeneous catalyst in 2‐methyl‐THF as a green solvent (Section S9). Full characterization of the zeolite has been performed in the prior work [36], and summarized in Table S7. Evolution of different products, quantified by HPLC (Figure S29), closely resembled what was measured by operando HSQC NMR in a deuterated system with dioxane and a homogeneous acid (Figure 3a), which confirms the applicability of the proposed reaction pathways to more practical conditions.

### 2.3 Reaction Path Analysis

**FIGURE 6:**
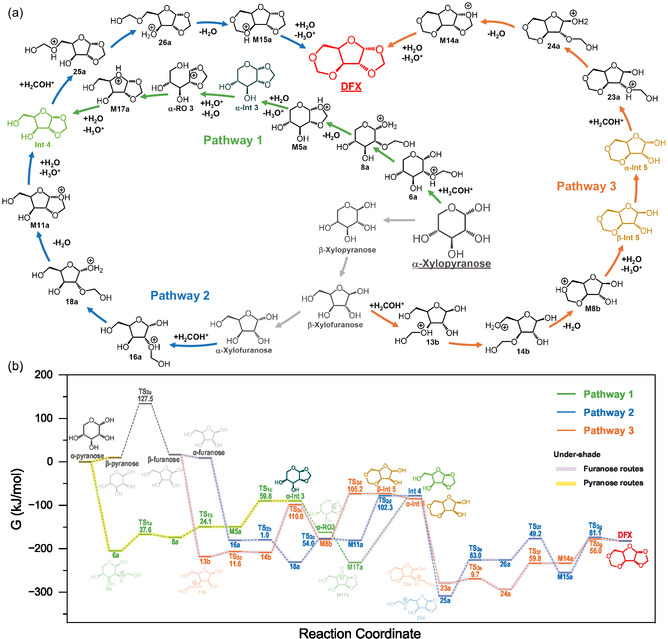
(a) DFT‐predicted lowest barrier pathways for acidic formylation of α‐D‐xylopyranose (*alpha* xylopyranose) to 5‐*O*‐diformylene‐D‐xylofuranose (DFX). (b) DFT‐calculated relative free energies (G, kJ/mol) of key reaction intermediates involved in the lowest barriers pathways shown in a,b for the conversion of *alpha* xylofuranose to DFX at 60°C. All transition states (TS) are denoted as TS_ij_, where ‘*i*’ denotes pathways (*i* =  1–3) and ‘*j*’ denotes the elementary step (*j* =  a‐g). Numbers denote the activation free energies (*G*_a_, kJ/mol) calculated as the formation free energy of the respective TS relative to the initial states from the left of the respective elementary steps. The elementary steps in (a) are colored to match the respective free energy diagrams in (b).

DFT calculations were performed to determine the energetically preferred pathways in the network proposed based on experimental results (Figure 5). We computed reaction free energies (Δ*G*) and activation free energies (*G*_a_) for over 140 possible elementary steps (Figures S19–S25) describing the acid‐catalyzed formylation of *α*‐D‐xylopyranose to 5‐O‐diformyl‐D‐xylofuranose (DFX). The lowest‐barrier pathways for the conversion of *α*‐xylopyranose to DFX are summarized in Figures 6 and S26. Comprehensive computational data, including zero‐point energies, entropies, and formation energies of individual intermediates, are provided in Table S5.

As validation of our computational framework, DFT‐calculated Δ*G* (kJ mol^−1^) for tautomerization of xylopyranose to xylofuranose agrees closely with experiments (Table S3): 9.7 (*α*) and 6.8 (*β*) for DFT versus 8.3 (*α*) and 6.8 (*β*) for experimental data. For its conversion to DFX, *α*‐D‐xylopyranose preferentially follows pathway 1: DFX formation through a pyranose monoacetal (**Int 3**), beginning with acetylation at the C2–OH to form intermediate **6a** (Δ*G* = −204.6 kJ mol^−1^, *G*_a_ =  0 kJ mol^−1^) (Figure 6). Subsequent intramolecular proton transfer from the C2‐ to the C1–OH (**6a** → **8a**) proceeds with a low activation free energy (*G*_a_ =  37.6 kJ mol^−1^). Intermediate 8a then undergoes dehydration at C1 and ring closure to form M5a (*G*_a_ =  24.1 kJ mol^−1^). The *α*‐anomer of the experimentally observed intermediate 3 (**Int 3** ***α***) forms via a moderate‐barrier deprotonation of M5a (*G*_a_ =  59.8 kJ mol^−1^). Int 3*α* exhibits a strong thermodynamic driving force (Δ*G* = −86.9 kJ mol^−1^) for reductive ring opening to generate the *α*‐anomer of the experimentally detected **RO 3** (**RO 3*α***) through protonation of the ring oxygen. RO 3*α* subsequently undergoes a thermodynamically favourable ring rearrangement to produce M17a (Δ*G* = −55 kJ mol^−1^), followed by a high‐barrier deprotonation step (Δ*G* =  153.4 kJ mol^−1^; M4b → **Int 4*α***) leading to the *α*‐anomer of the experimentally observed **Int 4**. The energetics of **Int 3*β*** formation from *β*‐xylopyranose and its subsequent conversion to **Int 4** follow the same trend, despite a slightly higher free energy of **Int 3*β*** than **Int 3*α*** (Δ*G* =  −47.3 vs. −89.7 kJ mol^−1^, Figures S20 and S23). Thus, the computation is consistent with the experimental observations of the formation of **Int 3** and its subsequent transformation to **Int 4** (Figure 4).

An alternative route to the experimentally observed Int 4 (Pathway 2, Figure 6) involves the energetically facile isomerization of *α*‐D‐xylopyranose to *β*‐D‐xylopyranose (Δ*G* = 9.7 kJ mol^−1^), consistent with previous reports of low‐barrier anomerization [37]. The subsequent water‐assisted tautomerization of *β*‐D‐xylopyranose to *β*‐D‐xylofuranose exhibits a relatively high activation free energy (*G*_a_ =  127.5 kJ mol^−1^), though it remains lower than the highest barrier in Pathway 1 (Δ*G* =  153.4 kJ mol^−1^). This large barrier likely arises from the inclusion of only a single explicit water molecule in our DFT model, which may underestimate the cooperative proton transfer facilitated by multiple water molecules. Based on prior studies [38], a lower tautomerization barrier is expected under experimental conditions. The resulting *β*‐D‐xylofuranose readily isomerizes to *α*‐D‐xylofuranose (Δ*G* = −7.7 kJ mol^−1^), which, analogous to Pathway 1, undergoes thermodynamically favourable formylation at C2–OH to generate intermediate 16a (Δ*G* = −189.1 kJ mol^−1^). Intermediate 16a subsequently undergoes a near‐barrierless intramolecular proton transfer from C2‐ to C1–OH (*G*_a_ =  1 kJ mol^−1^), followed by dehydration and ring closure to form M11a (18a → M11a, *G*_a_ =  54 kJ mol^−1^). The ensuing deprotonation step (M11a → Int 4*α*) presents a high barrier (*G*_a_ =  102.3 kJ mol^−1^). Overall, the apparent barrier—defined as the free‐energy difference between the lowest and next‐highest states—amounts to 152.5 kJ mol^−1^ for Pathway 2 (18a → Int 4*α*) and 153.4 kJ mol^−1^ for Pathway 1 (17a → Int 4*α*), indicating a slightly higher preference for Pathway 2 in forming Int 4*α*. Compared to Int 4*α*, Int 4*β* has a higher apparent barrier (186.2 kJ mol^−1^, M12b → Int 4*β*) due to higher ring strains caused by the trans‐positioned contiguous C1–O and C2–O bonds of the dioxolane ring, which is fused with the furanose ring (Figure S22). This difference explains the lack of any observed Int 4*β* or *β*‐xylose‐based DAX in reactions of xylose with various aldehydes (Figures 3 and 4). A mechanistically analogous route (Pathway 3) leads to the formation of Int 5*α* with an apparent barrier of 145 kJ mol^−1^. A more exergonic formation free energy of Int 5*α* compared to Int 4*α* (see Figure 6 and S27) explains the preferential formation of Int 5 over Int 4 in experiments with a less electrophilic aldehyde (e.g., propionaldehyde and dodecanal in Figure 3b,c).

The formation of DFX from **Int 4*α*** proceeds preferentially via the thermodynamically favourable formylation of the –CH_2_OH group (i.e., C5–OH) (Δ*G* = −230.6 kJ mol^−1^), followed by a high‐barrier intramolecular proton transfer (25a → 26a, *G*_a_ =  83 kJ mol^−1^), a low‐barrier dehydrative ring closure (26a → M15a, *G*_a_ =  29.2 kJ mol^−1^), and a subsequent high‐barrier deprotonation (*G*_a_ =  81.1 kJ mol^−1^). In contrast, Int 5*α* undergoes formylation at the C2–OH position, ultimately yielding DFX through a single dehydrative ring‐closure step (24a → M14a, *G*_a_ =  59.8 kJ mol^−1^), though this represents a limited reaction pathway. Other experimentally detected intermediates, including **Int 1** and **Int 2**, are predicted to form reversibly through formylation of the C3‐ and C2–OH of xylopyranose, respectively (Figure S26).

Overall, our DFT results corroborates the reaction intermediates detected by operando HSQC spectroscopy, indicating that DFX formation proceeds through three parallel pathways: Pathway 1 (*α*‐D‐xylopyranose ↔ 6a → 8a → M5a → **Int 3*α*** → **RO 3*α*** → M17a → **Int 4*α*** ↔ 25a → 26a → M15a → DFX); Pathway 2 (*α*‐D‐xylopyranose ↔ *β*‐D‐xylopyranose → *β*‐D‐xylofuranose ↔ *α*‐D‐xylofuranose ↔ 16a → 18a → M11a → **Int 4*α*** ↔ 25a → 26a → M15a → DFX); and Pathway 3 (*α*‐D‐xylopyranose ↔ *β*‐D‐xylopyranose → *β*‐D‐xylofuranose ↔ 13b → 14b → M8b → **Int 5*β*** → **Int 5*α*** ↔ 23a → 24a → M14a → DFX) (Figure 6a). Finally, noting the high computational cost of DFT calculations to evaluate the energetics of all elementary steps for other aldehydes, we instead performed additional calculations to compare the formation free energies of key intermediates (Int 1–5) involved in xylopyranose acetalization with formaldehyde and the less electrophilic propionaldehyde (Figure S27). The formation free energies of all propionaldehyde‐based intermediates were consistently more endergonic than those of the corresponding formaldehyde‐based species. These results confirm that aldehyde electrophilicity affected the yield or absence of specific xylose acetalization intermediates through differences in product stability.

## 3 Conclusion

Based on experiments and *ab initio* calculations, we propose a generalizable reaction network of xylose acetalization with various aldehydes. Two important factors govern cyclic acetal yield and selectivity: the aldehyde electrophilicity and the ring strain of the resulting cyclic acetals. The geometric orientations of xylose hydroxyls impose energetic constraints on ring closure, where trans‐positioned vicinal hydroxyls generate higher ring strains upon cyclization than cis‐positioned pairs, raising the energy barrier and reducing the likelihood of the formation of the corresponding acetals. Strongly electrophilic aldehydes like formaldehyde can compensate for these barriers through more favourable transition‐state stabilization, enabling acetalization of both furanose and pyranose. In contrast, aldehydes with weaker electrophilicity can only form less‐strained, cis‐positioned furanose acetals, as the combination of the low carbonyl electrophilicity and the higher fuzed‐ring strain of pyranose acetals renders those pathways energetically inaccessible.

Regardless of the pathways and aldehydes, furanose diacetals were always found to be the most abundant products given enough reaction time, which was rationalized by *ab initio* calculations as these furanose products were the most energetically favoured. The dominance of xylopyranose in the reactants and the contrasting dominance of furanose acetals in the products stress the importance of the pyranose‐furanose isomerization in xylose acetalization. The identified intermediates, combined with a mechanistically explained reaction network, could be used to identify potential products of interest and optimize their production. In addition, some of the principles outlined above can hopefully form a basis to study acetalization of other sugars.

## 4 Experimental Section

### 4.1 Reaction Kinetic Measurement

d‐xylose (50 mg, 1 mol. eq.) was predissolved in MQ water (0.25 mL), and the mixture was added to 1,4‐dioxane (5 mL) with the aldehyde of interest (2 mol. eq.) and H_2_SO_4_ (80 µL) in a 10 mL glass reactor. The mixture was heated to the reaction temperature of 60°C. A 0.1 mL aliquot was collected using a 0.1 mL microsyringe at each time point. The samples were diluted to 1 mL and neutralized with 0.1 g NaHCO_3_ before being filtered with 0.2 µm PTFE syringe filters. The DAX yield was quantified by gas chromatography (Agilent 7890B with an HP5 column and an FID). The residual xylose was quantified by HPLC (Agilent Infinity 1260 equipped with a Pursuit XRs C18 column using 10 vol.% acetonitrile in water as the eluent). Dioxane was used in all experiments to be consistent with the NMR solvent. Although green solvents like 2‐Methyl‐THF work equally well for synthesis [9], they are not commercially available in a deuterated form.

### 4.2 Operando NMR for Xylose Acetalization

Operando NMR spectra (^1^H and ^1^H‐^13^C HSQC) were acquired using a Bruker Avance 500 MHz spectrometer (11.75 T) with a 5 mm proton‐optimized triple resonance NMR “inverse” TCI Cryoprobe. The quantitative ^1^H spectra were recorded using a Hahn‐echo sequence with the recycle delay selected to be 11 s and ns =  8. ^1^H‐^13^C HSQC spectra were recorded using an optimized sequence with ns =  1 to attain a good resolution. The acquisition time for a ^1^H and a ^1^H‐^13^C HSQC spectrum was 2.5 and 3.5 min, respectively, to achieve a total of 6 min per set of data points. d‐xylose (5 mg, 1 mol. eq.) was predissolved in D_2_O (25 µL). 1,4‐dioxane‐d8 (0.5 mL) was mixed with various aldehyde (2 mol. eq.) and H_2_SO_4_‐d2(8 µL) in a high‐pressure NMR tube (New Era Enterprises, USA). The xylose solution was added to the NMR tube immediately before injecting the sample into the NMR spectrometer. The reaction was conducted at 60°C for 10 h, during which lock channel stability was maintained. The cross‐peak volumes in HSQC spectra were used to semi‐quantitatively track the evolution of various reactants, intermediates, and products. The integration regions were kept unchanged among all spectra to ensure comparable results. Quantitative 1H spectra were not used due to the many overlapping peaks. The reliability of the HSQC results was verified using the time‐zero HSQC method (see Section S6) [39, 40].

### 4.3 d‐xylose Tautomer Ratio Measurement

The operando ^1^H NMR spectra were acquired using a Bruker Avance 500 MHz spectrometer (11.75 T) with a 5 mm proton‐optimized triple resonance NMR “inverse” TCI Cryoprobe. The quantitative ^1^H spectra were recorded using a Hahn‐echo sequence with the recycle delay selected to be 5 T1 and ns =  32. High‐purity d‐xylose (pharmaceutical secondary standard, 25 mg) was predissolved in D_2_O (25 µL). The mixture was then diluted with 1,4‐dioxane‐d8 (0.5 mL) in a high‐pressure NMR tube (New Era Enterprises, USA). The NMR tube was pressurized with 5 bar N_2_ before the temperature ramping to avoid vigorous boiling in the tube. The NMR spectra were recorded from 20 to 140°C with 10°C increments. The sample was allowed to equilibrate for 2 h at each temperature before spectrum acquisition. The respective peak areas were fitted and integrated using Dmfit 2019 [41]. The ^1^H chemical shifts and *J*_HH_‐coupling constants of xylose tautomers were measured in D_2_O using a Bruker AvanceII 800 MHz spectrometer (18.79 T) with a 5 mm CPTCI_z_ Cryoprobe. A Hahn‐echo sequence was used with the recycle delay selected to be 5 T1 and ns =  16.

### 4.4 Ab Initio Calculations

All *ab initio* calculations were performed using the Gaussian 09 (revision D.01) software packages. [42] The geometries of all the intermediates were optimized at the M06‐2X level of theory with the 6‐311+g(d, p) basis set [43, 44, 45]. Single‐point calculations were performed for the optimized structures with the M06‐2X functionals and def2‐TZVP basis sets to accurately calculate the electronic properties [45, 46]. These methods have been used in previous *ab initio* calculations related to sugar derivatives and have been proven reliable [47, 48]. The convergence criteria for geometry optimization were selected as Max Force =  0.00045 Hartree/Bohr, RMS Force =  0.0003 Å, Max Displacement = 0.0018 Hartree/Bohr, and RMS Displacement =  0.0012 Å.

Activation free energies (*G*_a_) were calculated using an explicit H_2_O and H_3_O^+^ molecule through the Berny optimization algorithm [49]. All transition states were identified as first‐order saddle points based on a single imaginary vibrational frequency. Since multiple water molecules are known to participate [38], we anticipate that our computational framework may overestimate *G*_a_. Benchmarking calculations were performed to model the implicit solvent environment of dioxane using the solvation model based on density (SMD) variant of the polarizable continuum model employing the integral equation formalism variant [49]. These calculations revealed a uniform solvation correction of approximately −0.5 eV for all key intermediates (Table S6), indicating no net effect on the reaction energetics due to the implicit solvent treatment and validating conclusions based on free energy estimates made in vacuum.

Therefore, all free energy extrapolations were performed in vacuum at a temperature (*T*) of 333.15 K. An isolated H_3_O^+^ ion was used as a reference for the free energies of proton addition steps through Equations (1) and (2).

(1) $$A+H_{3}O^{+}\to B+H_{2}O$$

(2) $$\Delta G=G_{B}+G_{H2O}-G_{A}-G_{H3O+}$$

Here, *G*_i_ denotes the formation free energy of the respective isolated species *i* in vacuum at 333.15 K. The formation free energy of intermediate *i* is calculated using Equation (3).

(3) $$G_{i}\;=\;E_{i}\;+\;{\mathrm{ZPE}}_{i}\;–\;T\times S_{i}\;–\;a\times (E_{\mathrm{alpha}\mathrm{xylofuranose}}\;{\mathrm{ZPE}}_{\mathrm{alpha}\mathrm{xylofuranose}}\;–\;T\times S_{\mathrm{alpha}\mathrm{xylofuranose}})\;–\;b\times (E_{\mathrm{HCHO}}\;+\;{\mathrm{ZPE}}_{\mathrm{HCHO}}\;–\;T\times S_{\mathrm{HCHO}})\;–\;c\times (E_{H2O}\;+\;{\mathrm{ZPE}}_{H2O}\;–\;T\times S_{H2O})\;–\;d\times (E_{H3O+}+{\mathrm{ZPE}}_{H3O+}–\;T\times S_{H3O+})$$

Here, *E*_i_ denotes the electronic energy, ZPE_i_ denotes the zero‐point energy, and *S*_i_ denotes the entropy of intermediate *i*.

The electronic properties of aldehydes (Table S4) were calculated using Equations (4)–(6) proposed by Parr, Szentpaly, and Liu [32].

(4) $$\mu =\frac{\varepsilon_{\mathrm{HOMO}}+\varepsilon_{\mathrm{LUMO}}}{2}$$

(5) $$\eta =\frac{\varepsilon_{\mathrm{LUMO}}-\varepsilon_{\mathrm{HOMO}}}{2}$$

(6) $$\mathrm{EI}=\frac{\mu^{2}}{2\eta }$$

Here, *µ* denotes electronegativity, *η* denotes chemical hardness, EI denotes electrophilicity index, *ε*_HOMO_ denotes the highest occupied molecular orbital energy, and *ε*_LUMO_ denotes the lowest unoccupied molecular orbital energy. The electrophilicity index of C in the ‐CHO group (EI_‐CHO_ in Table S4) was calculated by multiplying EI from Equation (5) with the spin density of C in the radical state of the respective aldehydes. The Fukui index of C in the ‐CHO group of respective aldehydes was calculated as the difference in the Mulliken charges in the radical and neutral aldehydes.

## Supporting Information

Additional supporting information can be found online in the Supporting Information section. Experimental details, sample characterization, quantification methods, and data analysis and calculations are provided in the supporting information. All experimental and simulation data used in this study are available in a Zenodo repository 10.5281/zenodo.15701978. The coordinates of the optimized molecular structures of reaction aldehydes and reaction intermediates obtained from the *ab initio* calculation are also provided in the Zenodo repository. **Supporting** **Fig.** **S1:** Xylose calibration curve on the reverse‐phase HPLC. **Supporting** **Fig.** **S2:** 1,2;3,5‐O‐dimethylidene‐*α*‐D‐xylofuranose (DFX) calibration curve on the GC‐FID. **Supporting** **Fig.** **S3:** 1,2;3,5‐O‐dipropylidene‐*α*‐D‐xylofuranose (DPX) calibration curve on the GC‐FID. **Supporting Fig. S4** **:** 1,2;3,5‐O‐didodecylidene‐*α*‐D‐xylofuranose (DDX) calibration curve on the GC‐FID. **Supporting Fig. S5** **:** Identification of ^1^H‐^13^C HSQC cross peaks of intermediates and products in operando NMR in the reaction of xylose and formaldehyde. Processed results are presented in Figure 3a.The cross peaks corresponding to the C4 of xylose and its subsequent intermediates were used to measure relative yields. **Supporting Fig. S6** **:** Identification of ^1^H‐^13^C HSQC cross peaks of intermediates and products in operando NMR in the reaction of xylose and propionaldehyde. Processed results are presented in Figure 3b.The cross peaks corresponding to the C2 of xylose and its subsequent intermediates were used to measure relative yields. **Supporting Fig. S7** **:** Identification of ^1^H‐^13^C HSQC cross peaks of intermediates and products in operando NMR in the reaction of xylose and dodecanal. Processed results are presented in Figure 3c. The cross peaks corresponding to the C2 of xylose and its subsequent intermediates were used to measure relative yields. **Supporting Fig. S8** **:** Identification of ^1^H‐^13^C HSQC cross peaks of intermediates and products in operando NMR in the reaction of xylose and benzaldehyde. Processed results are presented in Figure 3d.The cross peaks corresponding to the C2 of xylose and its subsequent intermediates were used to measure relative yields. **Supporting Fig. S9** **:** Identification of ^1^H‐^13^C HSQC cross peaks of intermediates and products in operando NMR in the reaction of xylose and 2‐floruobenzaldehyde. Processed results are presented in Figure 3e. The cross peaks corresponding to the C2 of xylose and its subsequent intermediates were used to measure relative yields. **Supporting** **Fig.** **S10:** Identification of ^1^H‐^13^C HSQC cross peaks of Int 3 in high‐resolution operando NMR in the reaction of xylose and formaldehyde. Processed results are presented in Figure 4. The acetalmethylene cross peak is not present due to the use of formaldehyde‐d2. The cross peaks corresponding to the C1 of xylose and Int 3 were used to measure relative yields. **Supporting Fig. S11** **:** Comparison of the ratios of different reaction intermediates in simulated mixtures calculated from regular HSQC and gradient‐selective HSQC0 sequence. **Supporting Fig. S12** **:** The molar fraction of xylose, intermediates, and products measured by operando NMR as a function of reaction time when reacting with formaldehyde and dodecanal with and without the T2 relaxation correction. Reactions were conducted at 60°C in dioxane‐d8 with 2:1 aldehyde to xylose molar ratio and 1.6 vol.% D2SO4. **Supporting Fig. S13** **:** (a) The percentage of total peak volume over the course of the reaction relative to the total volume at the first time point. (b) Furfural yield during operando NMR xylose acetalization with PA and DA, quantified using quantitative operando ^1^H NMR with 1,2,4,5‐tetrachloro‐3‐nitrobenzene as the external standard. (c) Comparison of total product yields obtained using operando HSQC (corrected) and HPLC quantifications of operando NMR xylose acetalization experiments run with FA after 10 h (the last time step in Figure 3a). **Supporting Fig. S14** **:** ^1^H‐^13^C HSQC peak assignment for d‐xylose tautomers at (a) 25°C and (b) 140°C. The sample was dissolved in D_2_O due to high solubility. Trace of acetonitrile was added as the reference. (c) The ^1^H‐^13^C HSQC of methyl‐d‐xylofuranoside, and (d) the ^1^H‐^13^C HMBC spectrum of d‐xylose33tautomers at 140°C in D_2_O. The cross peaks linking the C1 peaks to the C2‐C4 peaks are highlighted in red. **Supporitng** **Fig.** **S15:** (a)‐(b) 1H and (c)‐(d) ^1^ ^3^C MR peak assignment for d‐xylose tautomers at 25°C in D_2_O. Chemicals shifts of pyranose are labelled in (a) and (c), while panels (b) and (d) zoom in to show the furanose peaks. *α*/*β*‐P denote *α*/*β*‐xylopyranose peaks and *α*/*β*‐F denote *α*/*β*‐xylofuranose peaks. **Supporting** **Fig.** **S16** **:** Linear regression of equilibrium constant for xylose isomerization reactions in 1,4‐dioxane, with C_i_/C_j_ being the tautomer concentration ratios and *1/T* being the inverse temperature. **Supporiting** **Fig.** **S17:** The summary of standard changes in enthalpy and entropy in xylose tautomerization in 1,4‐dioxane. Supporting Fig. S18: Correlation between the equilibrium pyranose monoacetal selectivity measured in the operando HSQC experiments and the electrophilicity index of the carbonyl carbon in different aldehydes used in Figure 3. The pyranose monoacetal selectivity is defined as selectivity (mol%) = $\frac{{\mathrm{Monoacetal}}_{\mathrm{pyranose}}}{{\mathrm{Monoacetal}}_{\mathrm{pyranose}}+{\mathrm{Monoacetal}}_{\mathrm{furanose}}}\times 100\%$. **Supporitng Fig. S19:** Elementary steps that were considered and the associated calculated reaction freeenergies (non‐underlined, ΔG (eV)) and activation free energies (underlined, G_a_ (eV)) for *α*‐Dxylopyranose(*alpha* xylopyranose) formylation to Int 1*α* (*alpha* Int1), Int 2*α* (*alpha* Int2), and Int3*α* (alpha Int3). **Supporting Fig. S20:** Elementary steps that were considered and the associated calculated reaction free energies (non‐underlined, ΔG (eV)) and activation free energies (underlined, G_a_ (eV)) for *β*‐Dxylopyranose(beta xylopyranose) formylation to Int 1*β* (beta Int1), Int 2*β* (beta Int2), and Int 3*β*(beta Int3). The cross sign indicates that a stable structure of the product involved in the corresponding elementary step was not found. Supporting Fig. S21: Elementary steps that were considered and the associated calculated reaction free energies (non‐underlined, ΔG (eV)) and activation free energies (underlined, G_a_ (eV)) for *α*‐Dxylofuranose(*alpha* xylofuranose) formylation to Int 4*α* (*alpha* Int4) and Int 5*α* (*alpha* Int5). The cross sign indicates that a stable structure of the product involved in the corresponding elementary step was not found. The barrier of the tautomerization step was derived in the presence of one explicit water molecule. **Supporting Fig. S22:** Elementary steps that were considered and the associated calculated reaction free energies (non‐underlined, ΔG (eV)) and activation free energies (underlined, G_a_ (eV)) for *β*‐Dxylofuranose(beta xylofuranose) formylation to Int 4*β* (beta Int4) and Int 5*β* (beta Int5). The cross sign indicates that a stable structure of the product involved in the corresponding elementary step was not found. The barrier of the tautomerization step was derived in the presence of one explicit water molecule. **Supporting** **Fig.** **S23:** Elementary steps that were considered and the associated calculated reaction free energies (ΔG (eV)) for the conversion of Int 3 to Int 4. **Supporting** **Fig.** **S24:** Elementary steps that were considered and the associated calculated reaction free energies (non‐underlined, ΔG (eV)) and activation free energies (underlined, G_a_ (eV)) for Int 4*α*(*alpha* Int4) and Int 5*α* (*alpha* Int5) formylation to DFX*α* (*alpha* DFX). The cross sign indicates that a stable structure of the product involved in the corresponding elementary step was not found. **Supporting** **Fig.** **S25:** Elementary steps that were considered and the associated calculated reaction free energies (non‐underlined, ΔG (eV)) and activation free energies (underlined, G_a_ (eV)) for Int 4*β*(beta Int4) and Int 5*β* (beta Int5) formylation to DFX*β* (beta DFX). The cross sign indicates that a stable structure of the product involved in the corresponding elementary step was not found. **Supporting** **Fig.** **S26:** Relative free energies (G, kJ/mol) calculated by DFT‐calculated for key reaction intermediates involved in the lowest barriers pathways for conversion of *alpha* xylofuranose to Int1 and Int 2 at 60°C. Numbers denote the activation free energies (G_a_, kJ/mol) relative to initial states from the left of the respective elementary steps. Notations are consistent with Figure S19‐S25. **Supporting** **Fig.** **27:** DFT‐calculated formation free energies (G, kJ/mol) of key reaction intermediates involved in the conversion of *alpha* xylofuranose to Int 1, Int 2, Int 3, Int 4, and Int 5 at 60°C. **Supporting** **Fig.** **28:** The mono‐ and diacetal product distribution of various monosaccharides’ acetalization with formaldehyde (FA) and dodecanal (DA). Reactions were conducted at 60°C for 4 h in dioxanewith a 2:1 aldehyde to xylose molar ratio and 1.6 vol.% H_2_SO_4_. Product yields were quantified using GC‐FID with effective carbon numbers as per our previous work. The proposed reaction pathways are also included. **Supporting** **Fig.** **S29:** The molar fraction of xylose, intermediates, and products as a function of reaction time when reacting it with formaldehyde as measured by HPLC. The reaction was conducted with HY zeolite as a heterogeneous Brønsted catalyst and in 2‐methylTHF as a greener alternative to 1,4‐dioxane (80°C with a 2:1 formaldehyde to xylose molar ratio. HY zeolite (SiO2:Al2O3 = 80:1) was loaded at 80 g/L solvent). Full characterization of HY zeolite can be found in prior work and summarized in Table S7. **Supporting** **Table** **1:** Summary of the spin‐spin relaxation constants and corrective factors of species in the formaldehyde reaction. **Suppor** **ting** **Table** **2:** Summary of the spin‐spin relaxation constants and corrective factors of species in the dodecanal reaction. **Supporting** **Table** **3:** Summary of linear regression data and the calculated thermodynamic parameters of xylose tautomerization in 1,4‐dioxane. **Supporting** **Table** **S4:** Electronic state properties of respective aldehydes calculated using M062X/def2‐TZVPlevel of theory. Notations: formaldehyde (FA), propanal (PA), dodecanal (DA), benzaldehyde(BnA), fluorobenzaldehyde (BnAF), highest occupied molecular orbital energy (HOMO), lowestoccupied molecular orbital energy (LUMO), electronegativity (μ), chemical hardness (η), and electrophilicity index of the molecule (EI), and electrophilicity index of the carbonyl carbon (EI_CHO_). **Supporting Table S5** **:** Zero‐point energies (ZPE), entropies (S), and formation free energies (G) calculated by DFT for reaction intermediates involved in acidic formylation of *α*‐D‐xylopyranose (*alpha* xylopyranose) to 5‐O‐diformylene‐D‐xylofuranose (DFX) at 333.15 K. Formation free energies are referenced to isolated HCHO, H_2_O, H_3_O^+^, and *α*‐D‐xylopyranose. **Supporting Table S6** **:** Solvation energies (Esolven_t_ – E_gas_) and free energies (G_solvent_ – G_gas_) for key reaction intermediates involved in acidic formylation of *α*‐D‐xylopyranose (*alpha* xylopyranose) to 5‐Odiformylene‐D‐xylofuranose (DFX) in 1,4‐dioxane at 333.15 K. **Supporting Table S7** **:** Characterization results of the HY zeolite used in this experiment.

## Funding

This work was supported by NCCR Catalysis (180544); National Centre of Competence in Research; Schweizerischer Nationalfonds zur Förderung der Wissenschaftlichen Forschung (TMCG‐2_213835).

## Conflicts of Interest

J.S.L. is a part owner of Bloom Biorenewables Ltd, a start‐up company that is commercializing the aldehyde functionalization chemistry of biomass‐derived molecules. The following authors are inventors in the patents related to acetal‐stabilized molecules: J.S.L., S.S., and Z.J.L. The remaining authors declare no competing interests.

## Supporting information

Supplementary Material

## Data Availability

The data that support the findings of this study are openly available in Zenodo at https://doi.org/10.5281/zenodo.15701978, reference number 15701978.
