# Supplementary material for: Aldehyde Electrophilicity and Ring Strain Govern Xylose Acetalization Pathways for Biobased Chemical Production
Source: ChemSusChem. 2025 Dec 29;19(1):e202501562. doi: 10.1002/cssc.202501562 (PMC12746704; doi:10.1002/cssc.202501562)
Supplement: Supplementary file 1 — Supplementary Material [file CSSC-19-e202501562-s001.pdf]

Aldehyde Electrophilicity and Ring Strain Govern Xylose Acetalization Pathways for Biobased Chemical Production

Supporting Information

Zezhong John Li,[a] Deep M. Patel,[b] Songlan Sun,[a] Claire Bourmaud,[a] Tso-Hsuan Chen,[b] Dionisios Vlachos,[b] Jeremy S. Luterbacher[a,*]

[a] *Laboratory of Sustainable and Catalytic Processing (LPDC), Institute of Chemicals Sciences and Engineering (ISIC), School of Basic Sciences (SB), École Polytechnique Fédérale de Lausanne (EPFL); Lausanne, Switzerland.*

[b] *Center for Catalytic Science and Technology and Delaware Energy Institute, Department of Chemical and Biomolecular Engineering, University of Delaware, 150 Academy St., Newark, DE 19716, USA.*

[*] Corresponding author: jeremy.luterbacher@epfl.ch

Contents

S1. Chemicals and materials 2

S2. Analytical methods 3

S3. Preparation and Characterizations 6

S4. Calibration 18

S5. HSQC peak identification 20

S6. Validation for HSQC semi-quantification 24

S7. Xylose tautomerization 30

S8. *Ab initio* calculations 35

S9. Acetalization of other monosaccharides 47

S10. Validation of Mechanistic Applicability under Practical Acetalization Conditions 50

Reference 52

S1. Chemicals and materials

All the commercial chemicals used in this study were of analytical grade and were used without further purification. Carl Roth AG supplied 1,4-dioxane (≥99.5%, stabilized with BHT), paraformaldehyde (granulated, extra pure), and benzaldehyde (≥99.5%). ABCR provided propionaldehyde (97%) and dodecanal (95%). Ivalua supplied 2-fluorobenzaldehyde (97%). Fisher Chemical supplied methanol (HPLC grade). Acros delivered acetonitrile (99.8%) as the reverse-phase HPLC eluent. Roth supplied sodium bicarbonate ($NaHCO_3$, 99%). Merck Millipore provided sulphuric acid (H_2SO_4, 95-97%, analytical) and ethyl acetate (≥99.5). Sigma-Aldrich supplied Dichloromethane (≥99.9%).

Most experiments were carried out using D-(+)-xylose (99%) purchased from Sigma-Aldrich. Exceptionally, D-(+)-xylose (Pharmaceutical Secondary Standard) from Sigma-Aldrich was used to measure the xylose tautomer ratio. NMR measurements were conducted using deuterated reactants and solvents. Zeotop supplied D_2O (≥99.9%), while CortecNet supplied 1,4-Dioxane-d8 (99.0%) and Sigma-Aldrich supplied D_2SO_4 (96-98 wt.% in D_2O, 99.5 atom % D) and formaldehyde-d2 solution (ca. 20 wt. % in D_2O, 98 atom % D). The internal standard for NMR quantification was 1,2,4,5-tetrachloro-3-nitrobenzene (quantitative NMR standard), and was purchased from Sigma-Aldrich.

Column chromatography was performed with silica gel (P60 40-63 µm, 60 Å, Irregular Silica Gels) obtained from Silicycle.

S2. Analytical methods

S2.1 Routine NMR

Routine NMR spectra (^1H, ^{13}C, ^1H-^{13}C HSQC, ^1H-^{13}C HMBC) were acquired using a Bruker Avance III 400 MHz spectrometer (T = 9.40 T) equipped with a $BBFO_z$ 5mm probe using the standard pulse sequences from Bruker. The purity of synthesized compounds is determined by quantitative 1H NMR with a recycle delay selected to be over 5 T1 using 1,2,4,5-Tetrachloro-3-nitrobenzene as internal standard.

S2.2 Operando HSQC

One-dimensional ^1H NMR spectra were inadequate to accurately measure the reaction intermediates and products as there were too many overlapping peaks. To separate the peaks in an additional dimension, a previously developed quick and sensitive (^1H-^{13}C) HSQC sequence was used to both gather useful information and perform operando experiments.[1] However, such HSQC measurements provided only semi-quantitative measurements due to variable T_2 relaxation rates across compounds. To verify that operando HSQC results were reasonably quantitative, we verified quantification of various compounds using the gradient-selective time-zero HSQC method (*vide infra*). For all operando NMR experiments, we used a Bruker Avance 500 MHz spectrometer (11.75 T) with a 5 mm proton-optimized triple resonance NMR 'inverse' TCI cryoprobe to reduce electronic noise and enhance acquisition sensitivity. A cryogenically cooled preamplifier also guaranties high lock and spectrometer stability even when running long experiments at high temperatures. Data collection was carried out using uniform sampling, with equal time increments in both ^1H and ^{13}C dimensions. Due to the high resolution and signal-to-noise ratio, we were able to reduce the number of HSQC scans to one, which allowed for frequent monitoring of the process.

To ensure sufficient signal intensity with a reasonable acquisition time, a recycle delay of 1.5 s was used.

S2.3 Gradient-selective $HSQC_0$ NMR

The volumes of cross-peaks in a traditional HSQC spectrum are not entirely quantitative due to the differing relaxation rates for different H—C bonds within the sample. On the other hand, the gradient-selective time-zero HSQC ($gsHSQC_0$) method offers quantitative extrapolation of NMR signals by correcting for the T_2 relaxation time leading to signal attenuation during the coherent transfer.[2,3] However, this method is not suitable for operando tracking of reaction intermediates as it typically takes hours to complete. Instead, $HSQC_0$ measurements were conducted on various isolated reactants, intermediates, and products to calculate their T_2 relaxation constants and to evaluate the reliability of the conventional HSQC sequence as a semi-quantitative approach (see SI, Section 6) . We used the same NMR setup for the operando and $HSQC_0$ experiments. A mixture of isolated analytes was added in 1,4-dioxane-d8, and the same sequence used in operando HSQC measurement was performed, followed by the $HSQC_0$ sequence. The details of the $HSQC_0$ pulse sequence were previously reported.[1,2]

Briefly, the strengths of the pulsed field gradients applied along the z-axis were g1: 80%, g2: 20.1%, g3: 60%, g4: 15.075%, g5: 40%, g6: 10.05% of the maximum of 53 G/cm, each with a duration of 1 ms followed by a 200 μs gradient recovery period. The sequences were run at 25°C, after 10 min of equilibration at this temperature. Shimming, matching and tuning were re-performed at high temperatures. The following experimental NMR parameters used were: Pulse program= $gsHSQC_{0,I}$, CNST2=145Hz, SW=13.5 ppm ^1H – 165.0 ppm ^{13}C, O1P = 6.75 ppm ^1H – 75.0 ppm ^{13}C, AQ = 0.08 s ^1H – 0.06s ^{13}C, D1 = 4-5 s, NS = 16, DS = 32.

S2.4 GC-MS

The Gas Chromatography-Mass Spectrometry (GC-MS) analysis was performed using an Agilent 7890B series GC instrument coupled with an HP5-MS capillary column and an Agilent 5977A series Mass Spectroscopy detector. The GC-MS method procedure involved the following steps: 1 μL of the sample was injected with an autosampler in split mode (split ratio: 25:1), and the injection temperature was set to 300°C. The column was initially kept at 40°C for 3 min, then heated at a rate of 30°C/min to 100°C, followed by a heating rate of 40°C/min to 300°C and held for 5 min. The injection temperature was 300 °C.

S2.5 GC-FID

The Agilent 7890B series GC equipped with an HP5 column and an FID was used to perform quantitative analyses of diacetals. The temperature ramping program was the same as that of the GC-MS. To quantify the results, external calibration standards were used, which were synthesized using the method outlined in section S3. Calibration curves were prepared while correcting for the purity of each isolated compound (see Figures S2-4), which was determined by quantitative 1H NMR (section S4).

S2.6 HPLC (C18 reverse phase chromatography)

The remaining xylose was analyzed using an Agilent Infinity 1260 High-Performance Liquid Chromatography (HPLC) system, equipped with a Pursuit XRs C18 column (150 x 10 mm, 5μm). The system used H_2O/ACN (90/10 vol.) as eluent at a flow rate of 2 mL/min and V_{inj} of 40 μL at 25°C. A Refractive Index Detector (RID) (G1362A) was used to detect the compounds. The quantification was done using external calibration standards with commercial xylose (Figure S1).

S3. Preparation and Characterizations

S3.1 General reaction conditions

D-xylose (50mg, 1 mol. eq.) was pre-dissolved in 0.25 mL of MQ water, and the resulting solution was added to 1,4-dioxane (5 mL) along with various aldehyde (2 mol. eq.) and H_2SO_4 (80 µL) in a 10 mL glass reactor with a perforated cap and a PTFE/silicone septum. The mixture was heated to 60°C. At each time point, a 0.1 mL aliquot was collected using a 0.1 mL microsyringe. The samples were diluted to 1 mL, neutralized with 0.1 g $NaHCO_3$, and filtered with 0.2 µm PTFE syringe filters. The microsyringe was cleaned with acetone and then 1,4-dioxane between each sample. The DAX yield was measured with GC-FID, while the residual xylose was quantified using reverse-phase HPLC.

S3.2 Operando NMR reaction conditions

D-xylose (5mg, 1 mol. eq.) was pre-dissolved in D_2O (25 µL) and kept aside. 1,4-dioxane-d8 (0.5 mL) was mixed with various aldehydes (2 mol. eq.) and H_2SO_4-d2(8 µL) in a high-pressure NMR tube (New Era Enterprises, USA). Immediately before loading the sample into the NMR spectrometer, the xylose-D_2O solution was added to the NMR tube. The reaction was performed at 60°C. The sample reached the reaction temperature within 10 min after loading, after which ^1H and (^1H-^{13}C) HSQC spectra were acquired periodically for 10h.

For the reactions with formaldehyde, a solution of 20 wt.% formaldehyde in D_2O was prepared. Paraformaldehyde (5.4g), D_2O (19.8 mL) and D_2SO_4 (8 µL) were constantly stirred at 45°C for 7 days until a clear solution was obtained. Other aldehydes were used as received from suppliers.

S3.3 Preparation of xylose-formaldehyde reaction intermediates

D-xylose (10 g, 1 mol. eq.), paraformaldehyde (2 g, 1 mol. eq.) and sulfuric acid (0.214 mL) were combined with 1,4-dioxane (200 mL) in a 500 mL round bottom flask with a condenser. The mixture was heated at 65°C for 3.5h. A NaOH aqueous solution (1M, 8 mL) was added to neutralize the acid after the reaction mixture was brought back down to room temperature. The mixture was concentrated in vacuo on a rotary evaporator with a bath temperature of 45°C. Most of the solvent was removed, and the mixture was re-dissolved in water and extracted twice with ethyl acetate. The organic layer was separated and dried on a rotary evaporator with a bath temperature of 45°C under reduced pressure (153 mbar). The residual mixture containing sugars, intermediate xylose monoacetals (MAXs), and the xylose diacetal (DAX), was dispersed with a small amount of dichloromethane. The MAXs were then separated from unreacted xylose and formaldehyde using column chromatography (ethyl acetate-hexane 30-100% gradient) to obtain a translucent pale gel. Despite repeated attempts to vary column packing and eluent compositions, individual MAXs could not be isolated using column chromatography due to their highly similar structures and strong polarity. Instead, a semi-preparative reverse-phase column (Pursuit XRs C18 column, 150 x 10 mm, 5µm) was used on an HPLC equipped with a fraction collector (Agilent 1260 Infinity G1364C) using H_2O/ACN (90/10) as the eluent (flow rate = 0.5 mL·min^{-1}, V_{inj} = 40 µL). The column temperature was kept at 25°C. Fractions corresponding to various peaks shown in RID were repeatedly collected. Respective fractions were combined and dried on a rotary evaporator with a bath temperature of 45°C under reduced pressure (20 mbar) to obtain small quantities of intermediates for identification using GC-MS and solution-state NMR.

One of the fractions, specifically the **Int 6** fraction (Figure 3) was found to be unstable during the drying process because of its less stable hemiacetal structure. To avoid this issue, a

gradual solvent replacement technique was used instead of direct drying on a rotary evaporator. The solvent mixture of H_2O/ACN was slowly replaced with D_2O at room temperature under reduced pressure (10^{-3} mbar). The solution was reduced to ca. 0.5 mL in vacuo before 1 mL of D_2O was added back to the mixture. This process was repeated 5 times until almost all solvent was replaced with D_2O. Then, this mixture was characterized with solution-state NMR and high-resolution mass spectrometry (nanochip-ESI/LTQ-Orbitrap).

S3.4 Preparation of other MAXs and DAXs

- MAXs synthesis

D-xylose (10 g, 1 mol. eq.), the aldehyde (1.5 mol. eq.) and sulfuric acid (0.214 mL, to make 0.02M) were added to 1,4-dioxane (200 mL) in a 500 mL round bottom flask with a condenser. The mixture was heated to 65°C for 5 h. The mixture was then cooled down to room temperature and an NaOH aqueous solution (1M, 8 mL) was added to neutralize the acid. The mixture was concentrated in vacuo on a rotary evaporator with a bath temperature of 45°C. Then the residue, which was viscous light yellow oil was washed with brine solution and extracted with EtOAc. The organic phase was then evaporated on a rotavap and purified using column chromatography (hexane-ethyl acetate) to obtain pale yellow solid MAXs. The compounds were characterized by GC-MS and solution-state NMR, and their purities were measured using quantitative ^{1}H NMR.

- DAXs synthesis

D-xylose (10 g, 1 mol. eq.), the aldehyde (2.1 mol. eq.) and sulfuric acid (2.14 mL, to make 0.2M) were added to 1,4-dioxane (200 mL) in a 500 mL round bottom flask with a condenser. The reaction was conducted at 80 °C for 3 h. Then, the solution was neutralized with 1 M NaOH

solution until the pH value became about 7. The solution was concentrated on a rotary evaporator with a bath temperature of 45 °C under reduced pressure (80 mbar). Then, the resultant viscous pale-yellow oil was washed with brine solution and extracted with hexane. The hexane phase was then concentrated to ca. 5 mL and put in the fridge at 5°C for crystallization. The product was collected by filtration and washed with cold ethanol to afford white crystals of DAXs. The compounds were characterized by GC-MS and solution-state NMR, and their purities were measured using quantitative ^{1}H NMR.

<u>S3.5 Int 3 detection and tracking</u>

D-xylose (0.1 g, 1 mol. eq.), D_2SO_4 (1 µL), and formaldehyde-d2 (20 wt.% in D_2O, 0.5 mL) were mixed in an NMR tube and vortexed until xylose was fully dissolved. The solution was then kept in a water bath at 45°C for 30 min. This resulting mixture was characterized using a Bruker Avance 500 MHz spectrometer (11.75 T) equipped with a 5 mm proton-optimized triple resonance NMR 'inverse' TCI cryoprobe at 20°C where spectra of ^{1}H, ^{13}C DEPT, (^{1}H-^{13}C) HSQC, and (^{1}H-^{13}C) HMBC were acquired using standard Bruker sequences.

25 µL of the above mixture was added to the 1,4-dioxane-d8 (500µL) with D_2SO_4 (8 µL). The new mixture was allowed to react at 60°C for 2h in the same NMR spectrometer. During the reaction, ^{1}H (ns = 16) and (^{1}H-^{13}C) HSQC (ns = 2) spectra were recorded every 18 min to track the conversion of various intermediates at the same reaction conditions as previous operando experiments. The transmitter offset frequency of the HSQC sequence was modified to 4.700 ppm for ^{1}H and 81.694 ppm for ^{13}C to improve the resolution, which led to a slightly longer acquisition time than the other operando HSQC sequences.

<u>S3.6 Xylose tautomer ratio measurement</u>

High-purity D-xylose (pharmaceutical secondary standard, 25 mg) was pre-dissolved in D_2O (25 µL). The mixture was then diluted with 1,4-dioxane-d8 (0.5 mL) in a high-pressure NMR tube (New Era Enterprises, USA). The NMR tube was pressurized with 5 bar N_2 before the temperature ramping to avoid vigorous boiling in the tube at high temperatures. The NMR spectra were recorded from 20°C to 140°C in 10°C increments. The sample was allowed to equilibrate for 2 h at each temperature before spectrum acquisition. The respective peak areas were fitted and integrated using Dmfit 2019.[4] The ^{1}H chemical shifts and J-coupling constants of xylose tautomers were measured using Bruker AvanceII 800 MHz spectrometer (18.79 T) with a 5 mm $CPTCI_Z$ Cryoprobe at 25°C. A Hahn-echo sequence was used with the recycle delay selected to be 5 T1 and ns=16. Acetonitrile was used as the standard for ^{1}H and ^{13}C chemical shift determination.

Despite the frequent documentation of xylopyranose,[5] we are not aware of the chemical shifts of xylofuranose having been reported previously. However, the ^{13}C NMR chemical shifts of methyl-d-xylofuranoside are available in the literature.[5] To provide additional confirmation for the chemical shift identification of xylofuranose, the chemical shifts of methyl-d-xylofuranoside were used as approximates, in addition to the ^{1}H, (^{1}H-^{13}C) HSQC, (^{1}H-^{13}C) HMBC, and ^{13}C DEPT NMR spectra. In detail, D-xylose (10 g, 1 mol. eq.), HCl (37 wt.%, 1 mL), and methanol (150 mL) were continuously stirred in a round bottom flask with a condenser at 55°C for 21 h. Excess $NaHCO_3$ was added to neutralize the acid before the mixture was filtered with celite. Methanol was removed using a rotary evaporator with a bath temperature of 40°C under reduced pressure (330 mbar). The residue was re-dissolved in dichloromethane to remove leftover salt. The organic mixture was then filtered with a 0.2 µm PTFE membrane filter and dried on a rotary evaporator with a bath temperature of 35°C under reduced pressure (380 mbar) to obtain a light brown viscous gel. The

compound was characterized by NMR and GC-MS, which confirmed it was methyl-d-xylofuranoside.

S3.7 Characterization data

S3.7.1 xylose tautomers

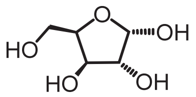

α-D-xylofuranose

^{1}H NMR (500 MHz, D_2O with 0.1% v/v acetonitrile, 298K) δ 5.42 (1H, d, *J* = 4.3, H1), 4.33 – 4.30 (1H, m, H3), 4.29 (1H, dd, *J* = 6.6, 3.0, H4), 4.11 (1H, t, *J* = 4.4, H2), 3.75 (1H, d, *J* = 3.7, H5'), 3.71 (1H, d, *J* = 3.1, H5").

^{13}C NMR (500 MHz, D_2O with 0.1% v/v acetonitrile, 298K) δ 96.0 (s, C1), 75.5 (s, C3), 78.9 (s, C4), 76.7 (s, C2), 61.0 (s, C5).

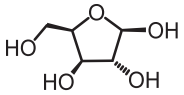

β-D-xylofuranose

^{1}H NMR (500 MHz, D_2O with 0.1% v/v acetonitrile, 298K) δ 5.22 (1H, d, *J* = 1.4, H1), 4.27 – 4.25 (1H, m, H4), 4.23 (1H, dd, *J* = 5.0, 2.3, H3), 4.08 (1H, t, *J* = 2.3, H2), 3.86 (1H, d, *J* = 4.4, H5'), 3.79 (1H, d, *J* = 4.7, H5").

^{13}C NMR (500 MHz, D_2O with 0.1% v/v acetonitrile, 298K) δ 102.2 (s, C1), 82.2 (s, C4), 75.5 (s, C3), 81.1 (s, C2), 61.3 (s, C5).

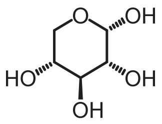

α-D-xylopyranose

^{1}H NMR (500 MHz, D_2O with 0.1% v/v acetonitrile, 298K) δ 5.18 (1 H, d, J = 3.7, H1), 3.68 (2H, d, J = 7.9, H5), 3.64 (1H, d, J = 9.1, H3), 3.64 – 3.58 (1H, m, H4), 3.51 (1H, dd, J = 9.4, 3.7, H2).

^{13}C NMR (500 MHz, D_2O with 0.1% v/v acetonitrile, 298K) δ 92.8 (s, C1), 61.5 (s, C5), 73.4 (s, C3), 70.0 (s, C4), 72.0 (s, C2).

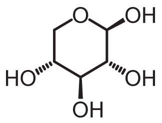

β-D-xylopyranose

^{1}H NMR (500 MHz, D_2O with 0.1% v/v acetonitrile, 298K) δ 4.56 (1H, d, J = 7.9, H1), 3.92 (1H, dd, J = 11.5, 5.5, H5'), 3.64 – 3.57 (1H, m, H4), 3.42 (1H, t, J = 9.3, H3), 3.31 (1H, t, J = 11.1, H5"), 3.21 (1H, dd, J = 9.4, 7.9, H2).

^{13}C NMR (500 MHz, D_2O with 0.1% v/v acetonitrile, 298K) δ 97.1 (s, C1), 65.7 (s, C5), 69.8 (s, C4), 76.3 (s, C3), 74.5 (s, C2).

S3.7.2 MAXs and DAXs

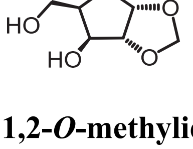

1,2-*O*-methylidene-α-D-xylofuranose (Int 4)

^{1}H NMR (400 MHz, $CDCl_3$) δ 6.00 (d, J = 3.7 Hz, 4H), 5.08 (s, 4H), 4.44 (d, J = 3.8 Hz, 5H), 4.40 (t, J = 3.3 Hz, 4H), 4.08 (m, 3H), 4.08 (d, J = 3.9 Hz, 3H), 4.04 (d, J = 2.6 Hz, 1H).

^{13}C NMR (101 MHz, $CDCl_3$) δ = 104.58, 96.60, 86.07, 80.48, 77.43, 61.49.

Silylated molecule GC-MS EI m/z: Calcd for $C_{12}H_{25}O_5Si_2^+$ 305.5; Found 306.0. Chromatogram reported in Zenodo.

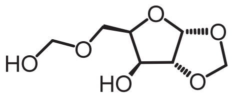

1,2-*O*-methylidene-5-*O*-methanol-α-D-xylofuranose (Int 6)

^1H NMR (500 MHz, D_2O) δ 6.04 (d, *J* = 4.2 Hz, 1H), 5.10 (d, *J* = 9.2 Hz, 5H), 5.06 (d, *J* = 6.4 Hz, 6H), 4.57 (d, *J* = 7.9 Hz, 2H), 4.37 (dd, *J* = 6.0, 3.1 Hz, 1H), 4.33 (dt, *J* = 7.3, 3.6 Hz, 1H), 4.05 (dd, *J* = 11.6, 5.4 Hz, 1H), 3.92 (dd, *J* = 11.6, 5.5 Hz, 2H).

^{13}C NMR (101 MHz, D_2O) δ = 103.83, 96.09, 91.88, 84.84, 81.33, 73.59, 64.45.

HRMS (nanochip-ESI/LTQ-Orbitrap) m/z: $[M + Na]^+$ Calcd for $C_7H_{12}NaO_6^+$ 215.0526; Found 215.0531.

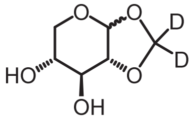

1,2-*O*-methylidene-D-xylopyranose (Int 3)

^1H NMR (500 MHz, D_2O, 20°C) δ 5.12 (d, *J* = 3.8 Hz, 1H), 4.56 (d, *J* = 7.8 Hz, 1H), 3.91 (dd, *J* = 11.5, 5.5 Hz, 4H), 3.71 (m, 3H), 3.69 (m, 1H), 3.64 (m, 2H), 3.57 (m, 1H), 3.49 (m, 2H), 3.42 (m, 2H), 3.30 (m, 1H), 3.27 (m, 2H).

^{13}C NMR (101 MHz, D_2O, 20°C) δ = 100.93, 96.49, 80.78, 78.50, 76.34, 73.56, 72.29, 65.70, 62.07.

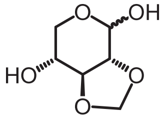

2,3-*O*-methylidene-D-xylopyranose (Int 2)

^{1}H NMR (400 MHz, $CDCl_3$) δ 5.33 (d, *J* = 3.6 Hz, 1H, 5), 5.08 (dd, *J* = 5.9, 3.9 Hz, 4H, 11''), 4.93 (dt, *J* = 6.2, 3.2 Hz, 4H, 11'), 4.71 (d, *J* = 7.6 Hz, 1H), 4.04 (dd, *J* = 11.6, 5.5 Hz, 1H, 1''), 3.83 – 3.80 (m, 2H, 2), 3.80 – 3.77 (m, 1H, 3), 3.77 – 3.73 (m, 1H), 3.56 – 3.50 (m, 1H, 4), 3.46 (t, *J* = 8.9 Hz, 1H), 3.36 – 3.31 (m, 1H, 1'), 3.29 (d, *J* = 4.2 Hz, 1H).

^{13}C NMR (101 MHz, $CDCl_3$) δ = 96.05, 92.11, 91.65, 83.50, 80.71, 80.54, 78.06, 68.51, 68.28, 60.94.

Silylated molecule GC-MS EI m/z: Calcd for $C_{12}H_{25}O_5Si_2^+$ 305.5; Found 306.0. Chromatogram reported in Zenodo.

α/β anomer: 60/40

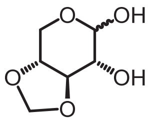

3,4-*O*-methylidene-D-xylopyranose (Int 1)

^{1}H NMR (400 MHz, $CDCl_3$) δ 5.26 (t, *J* = 3.4 Hz, 1H), 4.99 (dd, *J* = 5.9, 1.9 Hz, 2H), 4.95 – 4.86 (m, 2H), 4.65 – 4.53 (m, 1H), 4.03 (dd, *J* = 11.6, 5.4 Hz, 1H), 3.83 (t, *J* = 11.0 Hz, 1H), 3.76-3.68 (m, 1H), 3.76-3.65 (m, 2H), 3.71-3.62 (m, 3H), 3.71-3.63 (m, 2H), 3.62-3.54 (m, 3H), 3.54-3.42 (m, 2H), 3.51-3.41 (m, 2H), 3.36 (m, 1H).

^{13}C NMR (101 MHz, $CDCl_3$) δ = 97.53, 92.22, 92.38, 83.03, 80.92, 76.26, 75.94, 73.45, 70.95, 64.50, 60.15.

Silylated molecule GC-MS EI m/z: Calcd for $C_{12}H_{25}O_5Si_2^+$ 305.5; Found 305.9. Chromatogram reported in Zenodo.

α/β anomer: 60/40

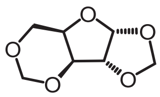

1,2;3,5-*O*-dimethylidene-α-D-xylofuranose (DFX)

^{1}H NMR (400 MHz, DMSO) δ 5.97 (d, *J* = 3.8 Hz, 1H), 4.98 (d, *J* = 5.8 Hz, 2H), 4.84 (d, *J* = 6.4 Hz, 1H), 4.63 (d, *J* = 6.4 Hz, 1H), 4.38 (d, *J* = 3.8 Hz, 1H), 4.30 (d, *J* = 2.3 Hz, 1H), 4.02 (d, *J* = 13.2 Hz, 1H), 3.93 (q, *J* = 2.0 Hz, 1H), 3.85 (dd, *J* = 13.2, 2.1 Hz, 1H).

^{13}C NMR (101 MHz, DMSO) δ = 103.99, 95.54, 90.31, 82.73, 76.60, 74.42, 64.78.

GC-MS EI m/z: Calcd for $C_7H_9O_5^+$ 173.1; Found 173.0. Chromatogram reported in Zenodo.

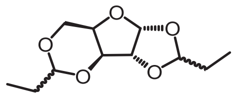

1,2;3,5-*O*-dipropylidene-α-D-xylofuranose (DPX)

^{1}H NMR (400 MHz, DMSO) δ 5.88 (d, *J* = 4.0 Hz, 1H), 4.87 (t, *J* = 4.5 Hz, 2H), 4.46 (t, *J* = 5.2 Hz, 2H), 4.37 (d, *J* = 4.0 Hz, 2H), 4.24 (d, *J* = 2.1 Hz, 3H), 4.12 – 4.01 (m, 1H), 3.93 (d, *J* = 8.3 Hz, 2H), 3.95 – 3.90 (m, 1H), 1.60 (dd, *J* = 7.5, 4.5 Hz, 4H), 1.52 – 1.42 (m, 4H), 0.85 (dt, *J* = 14.9, 7.5 Hz, 2H).

^{13}C NMR (101 MHz, DMSO) δ = 104.59, 104.29, 99.69, 83.36, 77.33, 71.84, 64.94, 27.01, 26.06, 7.86, 7.16.

GC-MS EI m/z: Calcd for $C_{11}H_{17}O_5^+$ 229.11; Found 229.2. Chromatogram reported in Zenodo.

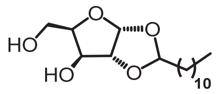

1,2-*O*-dodecylidene-α-D-xylofuranose (Int 4)

^{1}H NMR (400 MHz, $CDCl_3$) δ 5.96 (d, *J* = 3.7 Hz, 1H), 5.17 (t, *J* = 4.7 Hz, 0.52H), 4.92 (t, *J* = 4.8 Hz, 0.48H), 4.49 (d, *J* = 3.6 Hz, 1H), 4.42 – 4.31 (m, 2H), 4.16 – 3.93 (m, 4H), 1.16 – 1.42 (m, 18H), 0.86 (t, *J* = 6.7 Hz, 6H).

^{13}C NMR (101 MHz, $CDCl_3$) δ 106.91, 105.62, 104.65, 104.55, 86.43, 86.22, 81.47, 78.79, 77.09, 76.96, 61.33, 61.20, 34.72, 34.09, 32.03, 29.76, 29.75, 29.73, 29.63, 29.62, 29.56, 29.54, 29.48, 29.46, 23.85, 23.65, 22.80, 14.24.

HRMS (nanochip-ESI/LTQ-Orbitrap) m/z: $[M + H]^+$ Calcd for $C_{17}H_{33}O_5^+$ 317.2323; Found 317.2318.

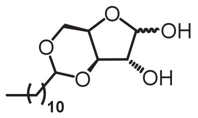

3,5-*O*-dodecylidene-xylose (MAX12, Int 5)

^1H NMR (400 MHz, $CDCl_3$) δ 5.69 (d, *J* = 3.8 Hz, 0.6H), 5.17 (s, 0.4H), 4.51-4.40 (m, 1H), 4.25 – 4.16 (m, 2H), 4.16 – 4.05 (m, 2H), 4.00 – 3.78 (m, 1H), 1.65-1.53 (m, 2H), 1.41 – 1.21 (m, 18H), 0.87 (t, *J* = 6.7 Hz, 3H).

^{13}C NMR (101 MHz, $CDCl_3$) δ 104.32, 100.52, 80.46, 73.94, 71.85, 67.40, 34.94, 34.83, 29.69, 29.64, 29.55, 29.47, 23.69, 22.80, 14.24.

Silylated molecule GC-MS EI m/z: Calcd for $C_{23}H_{47}O_5Si_2^+$ 459.3; Found 459.3. Chromatogram reported in Zenodo.

α/β anomer: 60/40

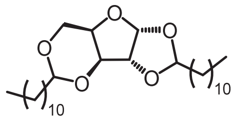

1,2;3,5-*O*-didodecylidene -α-D-xylofuranose (DDX)

^1H NMR (400 MHz, $CDCl_3$) δ 6.01 (d, *J* = 3.8 Hz, 1H), 4.94 (t, *J* = 4.8 Hz, 1H), 4.51 – 4.39 (m, 2H), 4.32-4.22 (m, 1H), 4.20 (d, *J* = 2.1 Hz, 1H), 4.02-3.98 (m, 1H), 3.96 – 3.88 (m, 1H), 1.72 – 1.55 (m, 4H), 1.43 – 1.17 (m, 36H), 0.87 (t, *J* = 6.98 Hz, 6H).

^{13}C NMR (101 MHz, $CDCl_3$) δ 105.48, 105.34, 100.57, 84.42, 78.48, 72.55, 66.22, 34.81, 34.77, 32.06, 32.05, 29.76, 29.68, 29.64, 29.62, 29.58, 29.51, 29.48, 29.39, 29.32, 29.21, 24.85, 23.97, 23.92, 23.80, 22.83, 14.26.

GC-MS EI m/z: Calcd for $C_{29}H_{53}O_5^+$ 481.4; Found 481.5. Chromatogram reported in Zenodo.

S4. Calibration

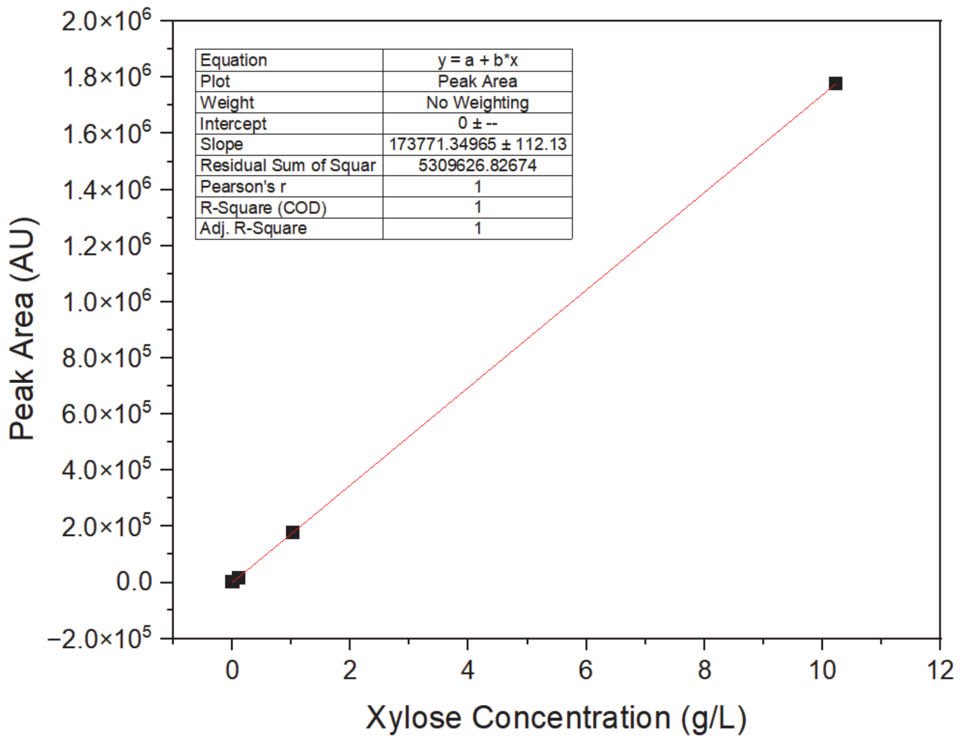

Figure S1. Xylose calibration curve on the reverse-phase HPLC.

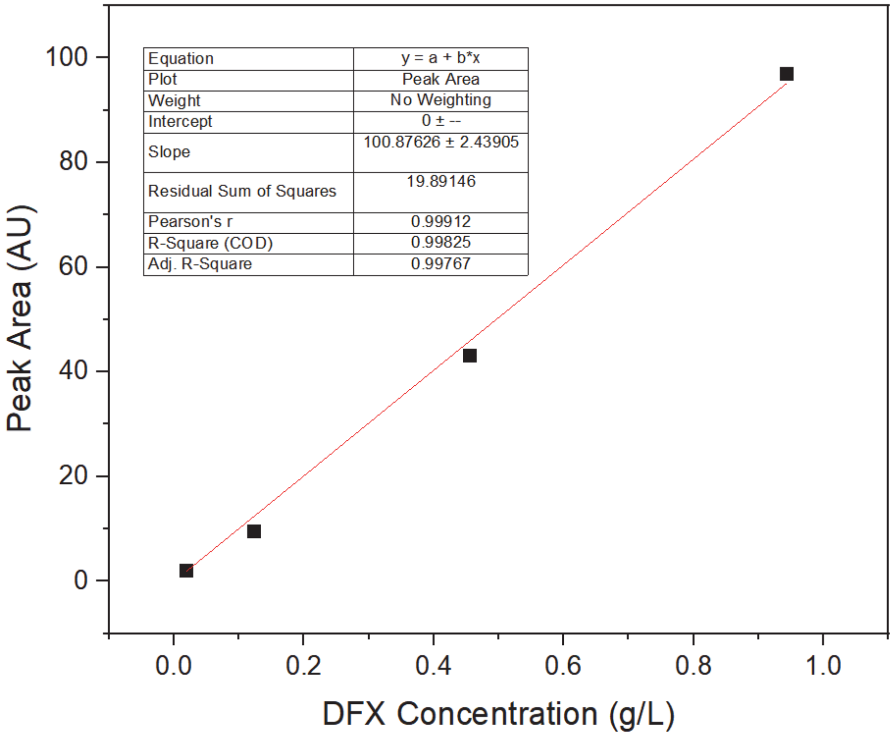

Figure S2. 1,2;3,5-*O*-dimethylidene-α-D-xylofuranose (DFX) calibration curve on the GC-FID.

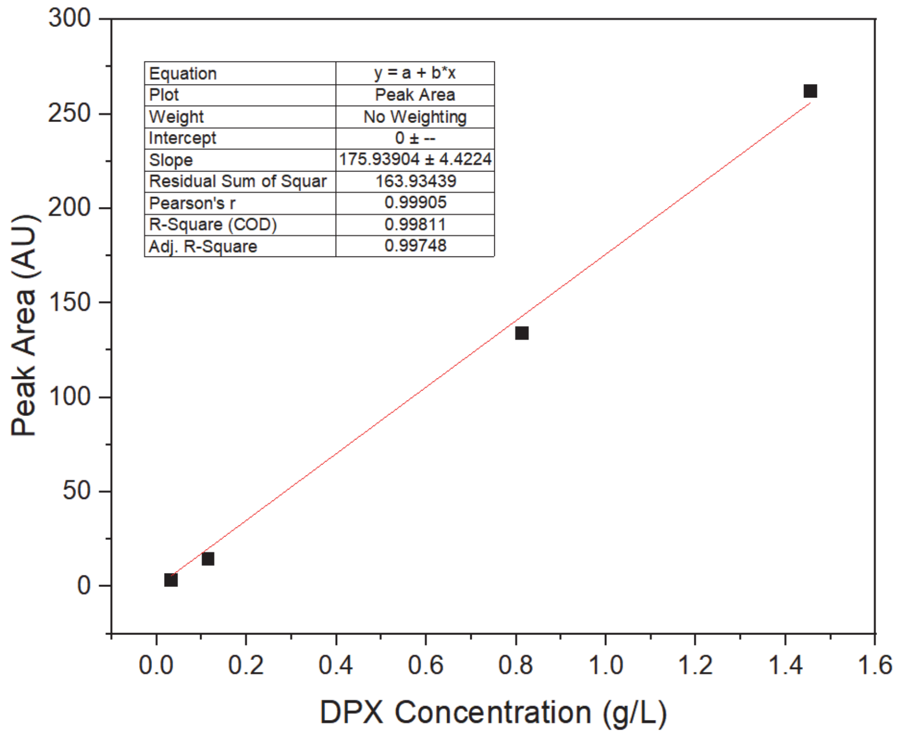

Figure S3. 1,2;3,5-*O*-dipropylidene-α-D-xylofuranose (DPX) calibration curve on the GC-FID.

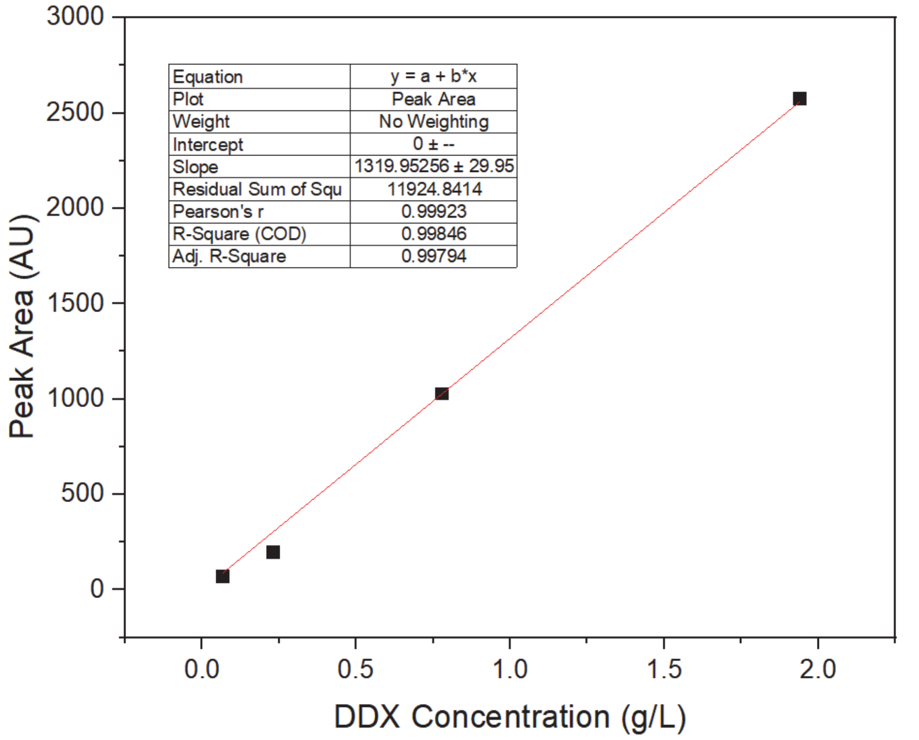

Figure S4. 1,2;3,5-*O*-didodecylidene -α-D-xylofuranose (DDX) calibration curve on the GC-FID.

S5. HSQC peak identification

The cross-peaks in the operando HSQC spectra were integrated using Bruker Topspin 4.1.3 to semi-quantitatively compute the molar ratio of each compound of interest. Figure S5-S9 illustrate the HSQC peak locations of these species in the reactions between xylose and formaldehyde, propionaldehyde, dodecanal, benzaldehyde, and 2-fluorobenzaldehyde, respectively. We tracked the cross-peaks corresponding to the same carbon-hydrogen pair in xylose and its subsequent mono- and diacetals for each reaction system. Fortunately, all the tracked cross-peaks were distinct with respect to each compound and did not significantly overlap with other peaks. The H-C4 cross-peaks were selected for the reaction with formaldehyde, while H-C2 cross-peaks were chosen for the remainder of the reactions. The numbering convention is shown in the respective figure with the tracked carbon highlighted.

Figure S10 highlights the HSQC peaks of **Int 3** using the different experimental procedure (*vide supra*). **Int 3** was quantified in reference to xylose based on the volume ratio of the respective peaks corresponding to C1. The other compounds were quantified based on the C4 of xylose, as in Figure S5. The molar fractions of these compounds are presented in Figure 4 in the main text.

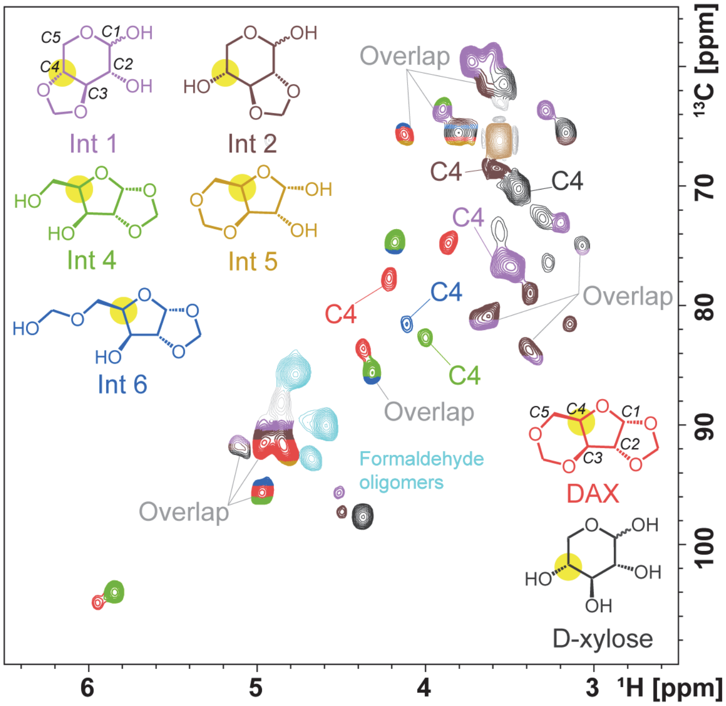

Figure S5. Identification of ^{1}H-^{13}C HSQC cross peaks of intermediates and products in operando NMR in the reaction of xylose and formaldehyde. Processed results are presented in Figure 3a. The cross peaks corresponding to the C4 of xylose and its subsequent intermediates were used to measure relative yields.

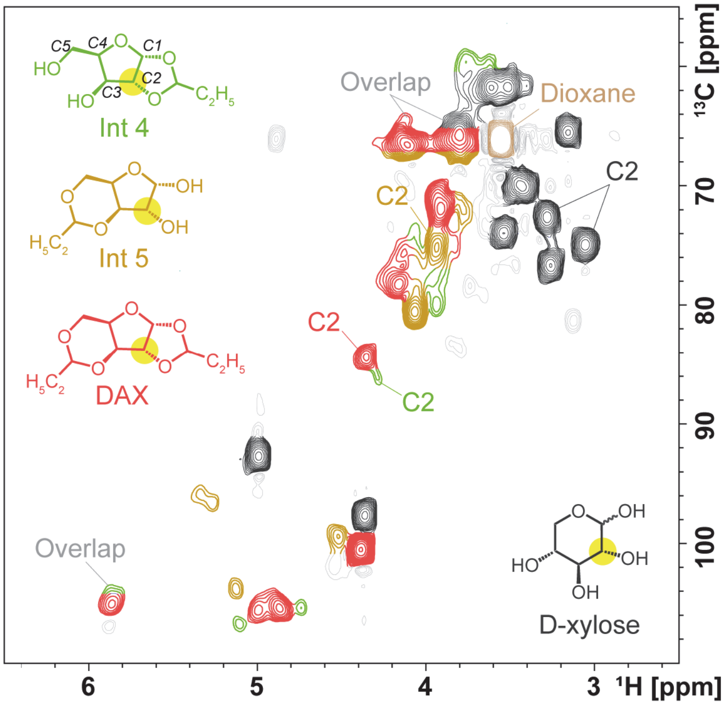

Figure S6. Identification of ^{1}H-^{13}C HSQC cross peaks of intermediates and products in operando NMR in the reaction of xylose and propionaldehyde. Processed results are presented in Figure 3b. The cross peaks corresponding to the C2 of xylose and its subsequent intermediates were used to measure relative yields.

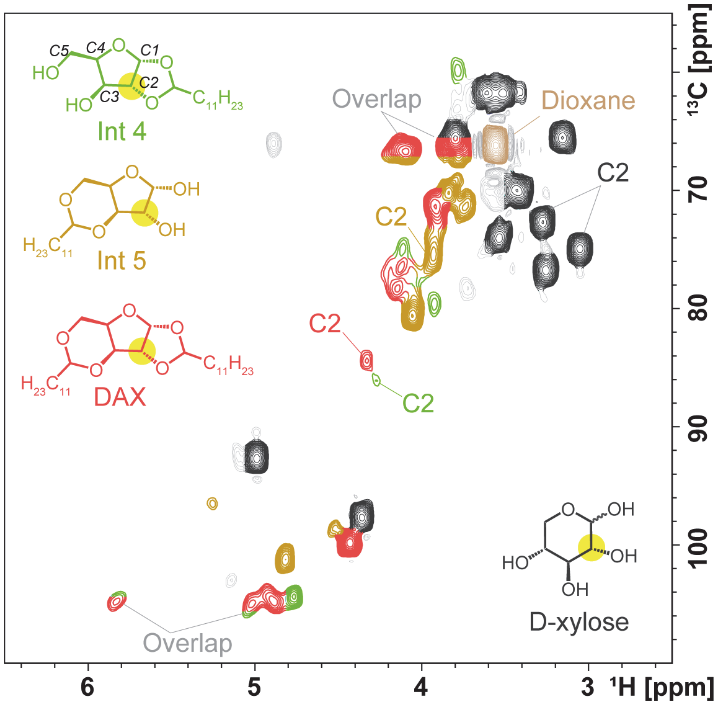

Figure S7. Identification of ^{1}H-^{13}C HSQC cross peaks of intermediates and products in operando NMR in the reaction of xylose and dodecanal. Processed results are presented in Figure 3c. The cross peaks corresponding to the C2 of xylose and its subsequent intermediates were used to measure relative yields.

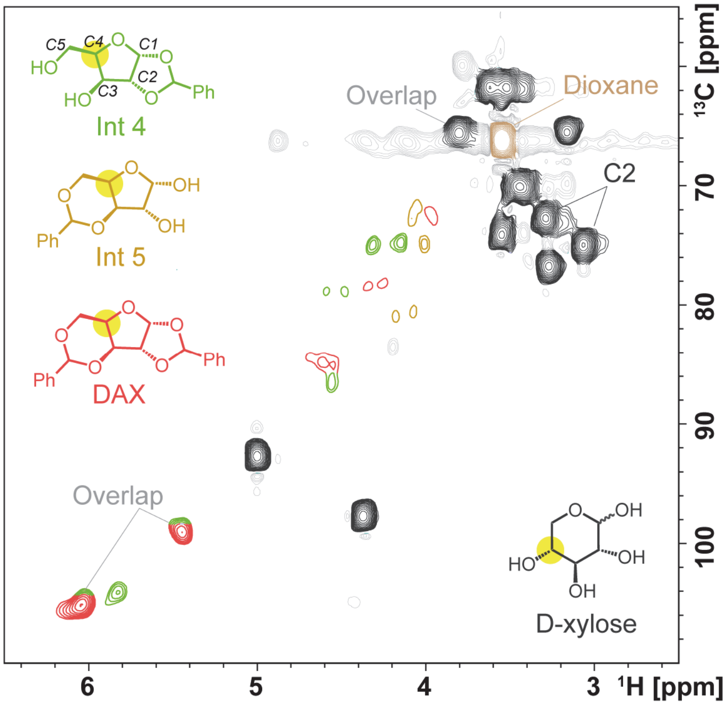

Figure S8. Identification of ^{1}H-^{13}C HSQC cross peaks of intermediates and products in operando NMR in the reaction of xylose and benzaldehyde. Processed results are presented in Figure 3d. The cross peaks corresponding to the C2 of xylose and its subsequent intermediates were used to measure relative yields.

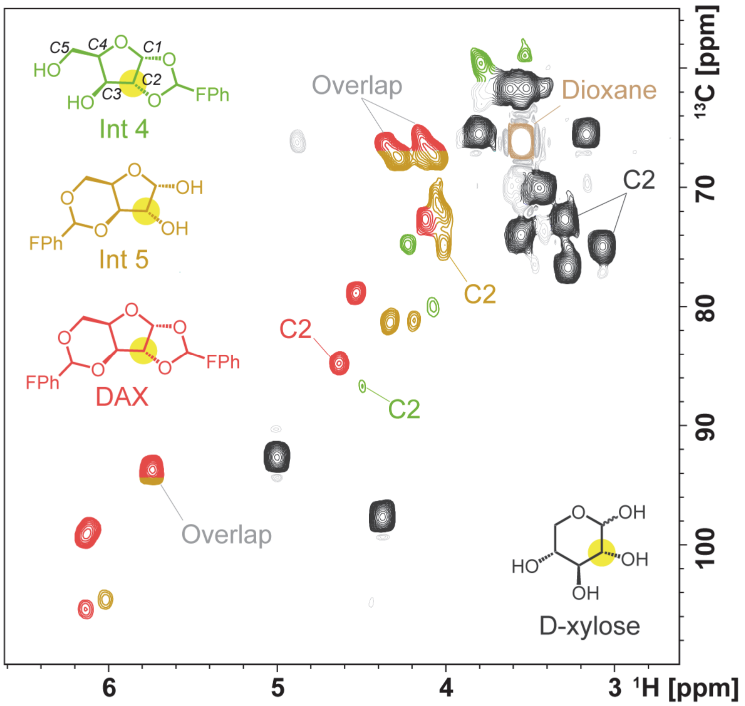

Figure S9. Identification of ^{1}H-^{13}C HSQC cross peaks of intermediates and products in operando NMR in the reaction of xylose and 2-floruobenzaldehyde. Processed results are presented in Figure 3e. The cross peaks corresponding to the C2 of xylose and its subsequent intermediates were used to measure relative yields.

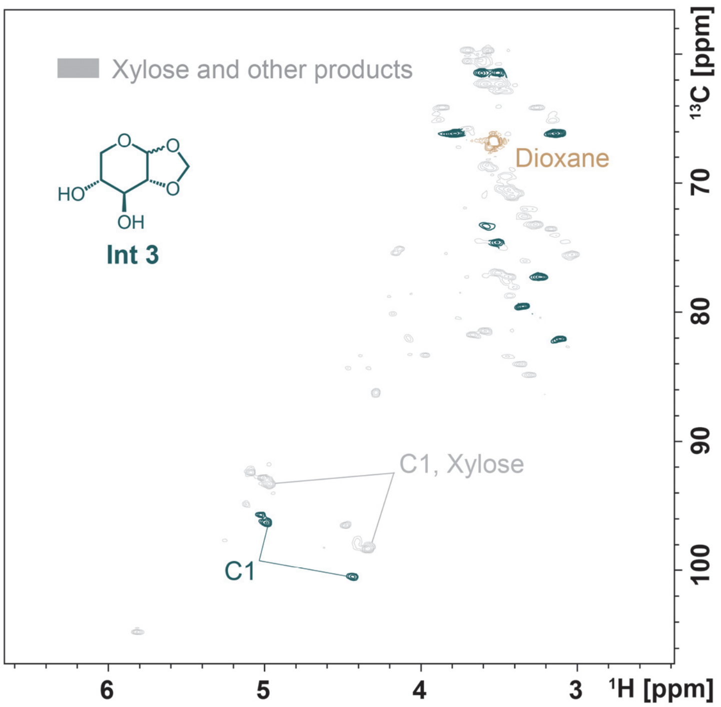

Figure S10. Identification of ^{1}H-^{13}C HSQC cross peaks of **Int 3** in high-resolution operando NMR in the reaction of xylose and formaldehyde. Processed results are presented in Figure 4. The acetal methylene cross peak is not present due to the used of formaldehyde-d2. The cross peaks corresponding to the C1 of xylose and **Int 3** were used to measure relative yields.

S6. Validation for HSQC semi-quantification

The cross-peak volumes in the operando HSQC spectra are not strictly quantitative due to the required relaxation time, so the ratios of different intermediates and products calculated from them are often considered semi-quantitative. To overcome this limitation, the gradient-selective time-zero HSQC method (gsHSQC$_0$) was used, which offered quantitative extrapolation of the NMR signals to time zero, compensation for signal attenuation during the coherent transfer due to T_2 relaxation.[3,6] However, HSQC$_0$ sequences are prohibitively long for operando measurements, so simulated reaction mixtures with various isolated constituents in the reaction mixture were used as a static snapshot of the reaction. To avoid reactions during HSQC$_0$ acquisitions, mixtures were kept at 25°C and no acid was added as the catalyst.

Even though the rate of T_2 relaxation was found to be dependent on temperatures and the ionic environment,[7,8] this method provided a preliminary assessment of these semi-quantitative results. We compared the ratio of each constituent in the simulated mixture, calculated using the HSQC spectra acquired with the same sequence as the operando measurements and those acquired using the HSQC$_0$ method (Figure S11). Almost all ratios in the operando measurements were within 5% of the more quantitative ratios calculated using the HSQC$_0$ method , demonstrating that the operando results provide quasi-quantitative molecular ratios. The spin-spin relaxation time, T_2 can be calculated using the slope of HSQC$_0$ regression (Eq. 1), which is then used to calculate the corrective factor, *F*, for each species to compensate for the T_2 relaxation in the operando HSQC sequence (Eq. 2):

$$T_2 = -\frac{t_{trans,\ HSQC_1}}{Slope} \tag{1}$$

$$F = \exp\left(\frac{t_{trans,\ operando}}{T_2}\right) \tag{2}$$

where $t_{trans,\ HSQC_1}$ is the unit total coherence transfer time immediately after the first ^{1}H excitation pulse till immediately before acquisition in the $HSQC_1$ sequence (47.401 ms), and $t_{trans,operando}$ is the total coherence transfer time in an operando HSQC sequence (44.318 ms). The calculated T_2 constants and the corrective factors are summarized in Table S1-S2.

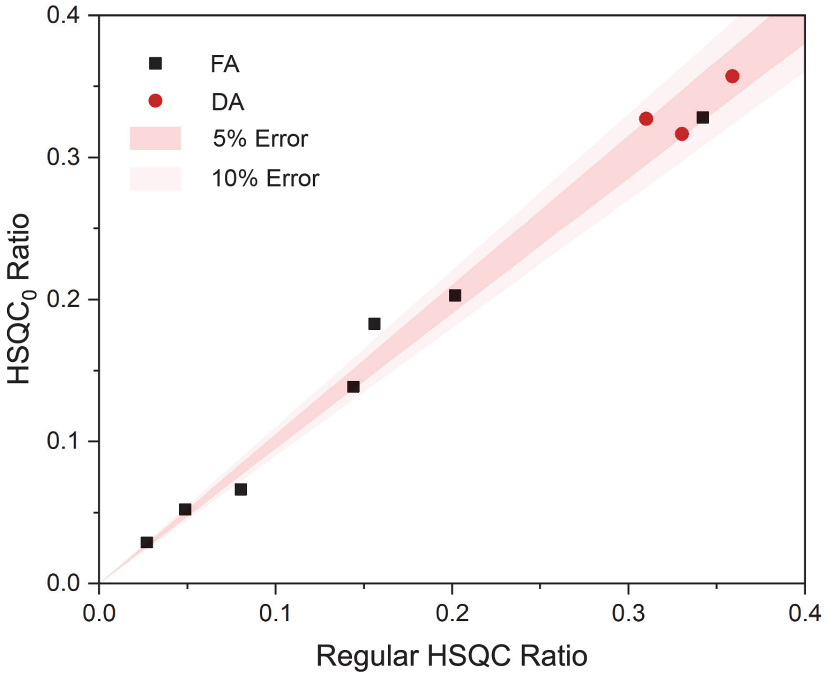

Figure S11. Comparison of the ratios of different reaction intermediates in simulated mixtures calculated from regular HSQC and gradient-selective $HSQC_0$ sequence.

Table S1. Summary of the spin-spin relaxation constants and corrective factors of species in the formaldehyde reaction.

	**Relaxation constant, T_2 (ms)**	**Corrective factor, F (-)**
**D-Xylose – C4**	55.56 ± 0.27	2.22 ± 0.01
**Int 1 – C4**	43.96 ± 0.59	2.74 ± 0.04
**Int 2 – C4**	44.37 ± 0.22	2.71 ± 0.01
**Int 4 – C4**	53.04 ± 0.12	2.30 ± 0.00
**Int 5 – C4**	54.12 ± 0.05	2.27 ± 0.00
**Int 6 – C4**	52.62 ± 0.27	2.32 ± 0.01
**DFX – C4**	58.92 ± 0.40	2.12 ± 0.01

Table S2. Summary of the spin-spin relaxation constants and corrective factors of species in the dodecanal reaction.

	**Relaxation constant, T_2 (ms)**	**Corrective factor, F (-)**
**D-Xylose – C2***	54.73 ± 0.47	2.25 ± 0.02
**Int 4 – C2**	58.25 ± 0.43	2.14 ± 0.01
**Int 5 – C2**	55.23 ± 0.30	2.23 ± 0.01
**DDX – C2**	56.64 ± 1.08	2.19 ± 0.03

* Average of xylopyranose anomers. Furanose signals were below detection limit.

The T_2 relaxation constants calculated from the $HSQC_0$ measurements are mostly around 50 ms, suggesting that the rotational correlation time of the molecules does not significantly change among all these intermediates and products.[9] HSQC signal attenuation would occur at similar rates for molecules with similar T_2. Despite the small difference between T_2, we further calculated the corrective factors to convert the signal intensity in a regular HSQC spectrum to those in an extrapolated $gsHSQC_0$ spectrum. As expected, these corrective factors are of similar values around 2.3 ± 0.2. The fractions of reactants, intermediates, and products were recalculated using the corrective factors during the formaldehyde and dodecanal reactions (Figure S12). The reliability of the semi-quantitative results obtained from the operando HSQC spectra was confirmed by the negligible differences obtained with the T_2 relaxation correction. Given these results, $HSQC_0$ was

not performed in the other systems involving propionaldehyde, benzaldehyde, and 2-fluorobenzaldehyde (see Figure 3 in the main text) due to the cumbersome sample preparation and long acquisitions. For consistency, all results shown in Figure 3 did not include T_2 correction, as its effect was shown to be negligible in the example systems of formaldehyde and dodecanal (Figure S12).

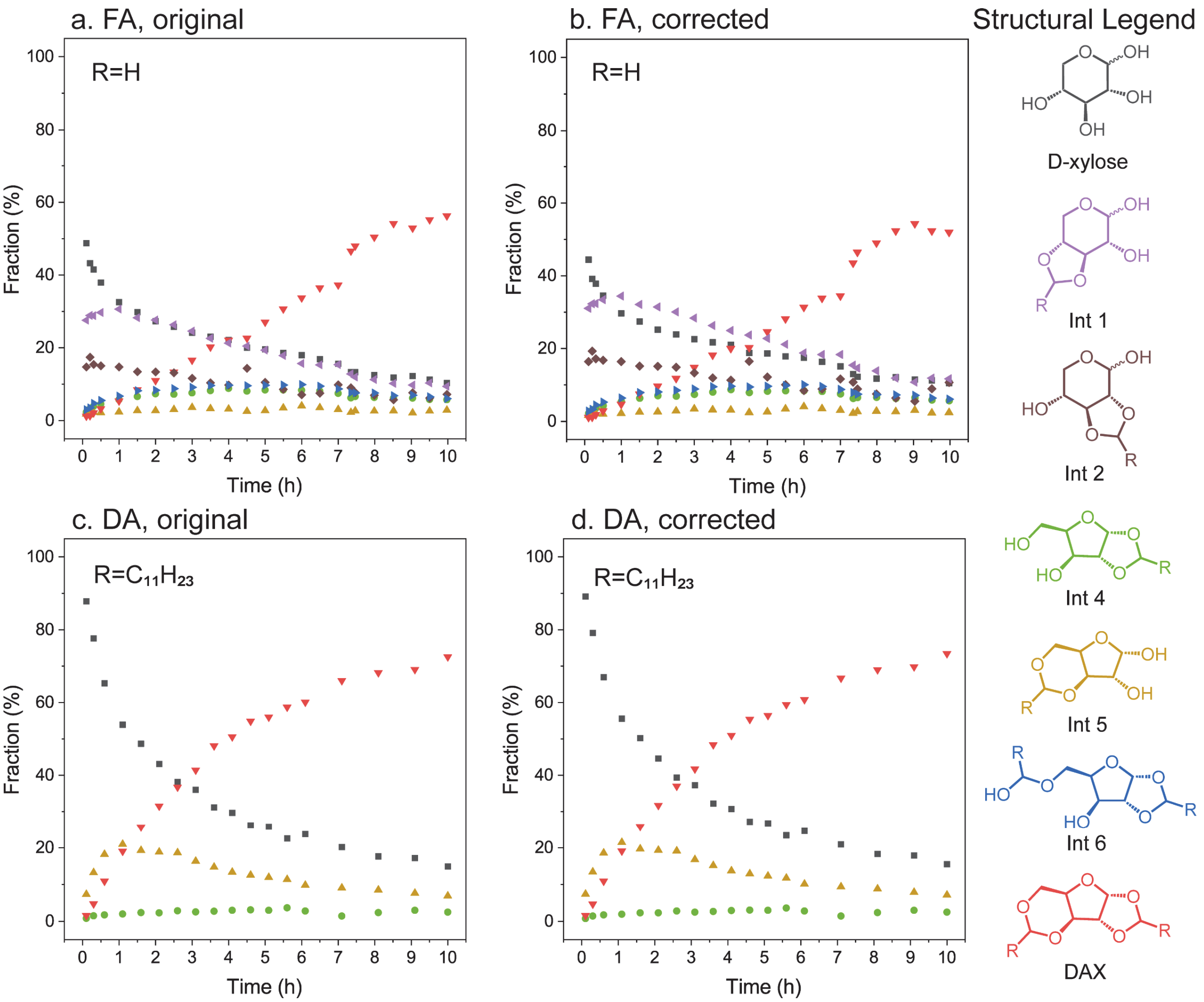

Figure S12. The molar fraction of xylose, intermediates, and products measured by operando NMR as a function of reaction time when reacting with formaldehyde and dodecanal with and without the T_2 relaxation correction. Reactions were conducted at 60°C in dioxane-d8 with 2:1 aldehyde to xylose molar ratio and 1.6 vol.% D_2SO_4.

In order to confirm that all major reaction intermediates and products were accounted for, the sum of the volume of the identifying peak of each compound was calculated and compared to the reference solvent peak volume. The ratio of total peak volume over the course of the reaction to the total volume at the first time point was then calculated (Figure S13a). For all the 5 reactions, the total volume slightly decayed over time but never more than 7% of the original volume over 10 h, which can be attributed to side reactions causing degradation and the T_2 relaxation time variation of the reaction intermediates. Nonetheless, the lack of any significant decay suggests that most of the reaction intermediates and products were systematically accounted for.

To directly assess possible carbon losses through dehydration or condensation, operando NMR measurements were repeated with two weaker aldehydes, pentanal (PA) and dodecanal (DA), using 1,2,4,5-tetrachloro-3-nitrobenzene as an external standard (sealed in a capillary inside the NMR tube). We monitored potential degradation products including furfural, formic acid, and acetic acid using quantitative ^{1}H NMR spectroscopy. Only trace furfural signals (<0.5% yield over 10 h; Figure S13b) were detected, while other degradation products were below the detection limit, confirming negligible carbon loss through dehydration or humin formation under the applied conditions.

Additionally, the endpoint sample from the 10 h operando NMR experiment of xylose acetalization with FA was analyzed by HPLC for independent quantification of xylose and its acetal products. The results closely matched the HSQC NMR data, with total molar balances exceeding 97% (97.7% by HPLC vs. 99.1% by HSQC; Figure S13c). This strong agreement confirms the reliability of the HSQC-based quantification and supports the conclusion that all major carbon-containing species were accurately measured throughout the reaction.

The low extent of degradation is further supported by the specific reaction conditions employed in the operando setup. The use of deuterated solvents and D_2SO_4 introduces a kinetic isotope effect,[10] resulting in slower cation-transfer reactions and suppressed side reactions compared to protiated systems. This effect is consistent with the slower xylose consumption observed in Figure 3 (deuterated systems) compared to Figure 2 (non-deuterated systems). Moreover, the reaction temperature was deliberately limited to 60°C to minimize dehydration.

Together, these findings confirm that dehydration and decomposition were negligible under the operando conditions (60°C, D_2SO_4, deuterated dioxane), validating the mass balance and supporting the conclusion that furanose diacetals are the predominant and stable end products.

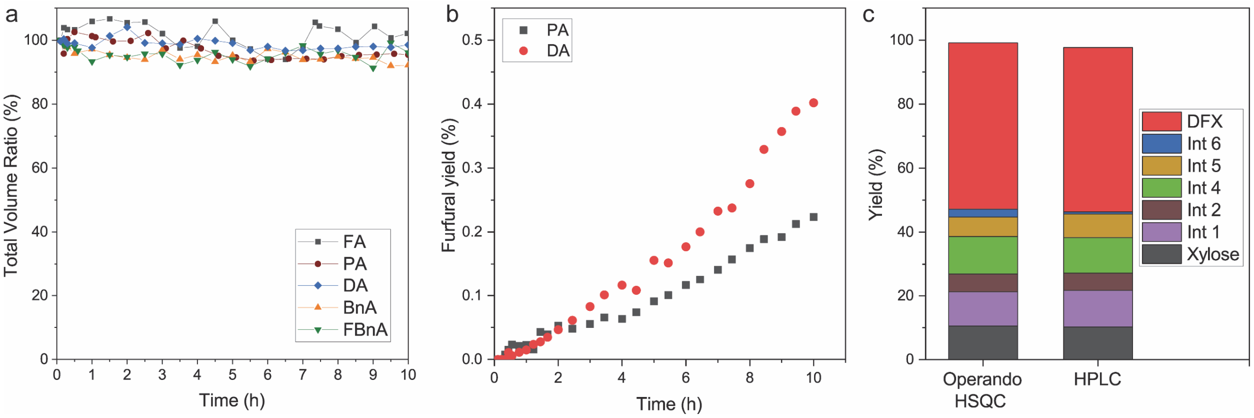

Figure S13. (a) The percentage of total peak volume over the course of the reaction relative to the total volume at the first time point. (b) Furfural yield during operando NMR xylose acetalization with PA and DA, quantified using quantitative operando ^{1}H NMR with 1,2,4,5-tetrachloro-3-nitrobenzene as the external standard. (c) Comparison of total product yields obtained using operando HSQC (corrected) and HPLC quantifications of operando NMR xylose acetalization experiments run with FA after 10 h (the last time step in Figure 3a).

S7. Xylose tautomerization

Aldoses, such as xylose and glucose, undergo well known tautomerization between their 4 cyclic isomers (i.e., α-furanose, β-furanose, α-pyranose, and β-pyranose) as well as their ring-opened linear aldehyde, albeit at a very low ratio (see main text and **Figure 1a**).[11] However, their equilibrium ratio heavily depends on the solvent and the temperature, and we are not aware of a report of the xylose isomer ratio at temperatures beyond the standard conditions. To weigh the significance of pyranose and furanose pathways and to be of reference for future xylose valorization studies, the molar ratio of each cyclic isomer of D-xylose between 20 and 140°C was measured using operando quantitative ^{1}H NMR in 1,4-dioxane-d8.

Notedly, the chemical shifts of xylofuranose anomers were not found in the literature, unlike xylopyranose anomers. Therefore, we had first to determine the chemical shifts of xylofuranose anomers to identify the corresponding peaks for quantitative calculations of all four cyclic tautomers. To improve the signal-to-noise ratio for furanose, ^{1}H, ^{13}C and (^{1}H-^{13}C) HSQC spectra were taken at 140°C, as the furanose isomer ratio increased with increasing temperature. Spectra were also recorded at 25°C for standard reporting. Highly pure D-xylose was used to avoid misidentification of impurity peaks. Chemical shift determination was conducted using D_2O in reference to acetonitrile peaks, as the solvent as xylose is more soluble in D_2O than 1,4-dioxane. Undocumented peaks were observed and their chemical shifts were close to the documented values for 1-methyl-*O*-D-xyloside, except for C1 (Figure S14c).[5] The HMBC spectrum confirmed the connectivity of these peaks (Figure S14d). The HSQC peak assignment is shown in Figure S14a-b, while the peak assignment in 1D ^{1}H and ^{13}C spectra is shown in Figure S15.

Quantitative ^{1}H NMR spectra were used to calculate the isomer ratio by integrating the areas of peaks corresponding to the C1 of xylose as they are the least overlapping peaks. Partially overlapping peaks of α-xylopyranose and β-xylofuranose were deconvoluted using Dmfit software.[4] Due to the small β-xylofuranose peak at low temperatures, the deconvoluted results could contain higher uncertainties than at high temperatures. These spectra were recorded for xylose dissolved in 1,4-dioxane with 5 vol.% water to best mimic the reaction conditions in this work. The calculated ratio of each isomer was used to calculate the equilibrium constant and the apparent standard change in enthalpy and entropy (ΔH°_{app} and ΔS°_{app}, respectively) of these isomerization reactions. The linear regression plots are shown in Figure S16, with the corresponding regression and calculation details summarized in Table S3. The standard changes in enthalpy and entropy between the cyclic tautomers of xylose are summarized in ascending order in Figure S17. Note that the ΔH°_{app} and ΔS°_{app} between the respective α/β anomers of furanose and pyranose have relatively large uncertainties due to small energetic and entropic changes during anomerization. On the other hand, the ΔH°_{app} and ΔS°_{app} between the furanose and pyranose were large enough to afford more reliable calculations. The linear isomer was not included in **Figure 1b** as it was never detected. Over the temperature range, xylose stayed predominantly in the pyranose form, despite the slight increase in xylofuranose as the temperature increased. The dominance of the pyranose structure is consistent with D-glucose.[11] The apparent standard reaction enthalpy and entropy were calculated as be 11.3 ± 1.0 kJ/mol and 13.6 ± 2.7 J/(K·mol), respectively, suggesting that the conversion from xylopyranose to xylofuranose is more entropically driven. The changes in enthalpy and entropy for the α-β anomerization are not reported here due to their relatively large experimental errors, as the changes in ratios with temperature are an order of magnitude smaller

compared to the furanose-pyranose isomerization (SI Section S7), which is consistent with the computational predictions for glucose anomerization.[12]

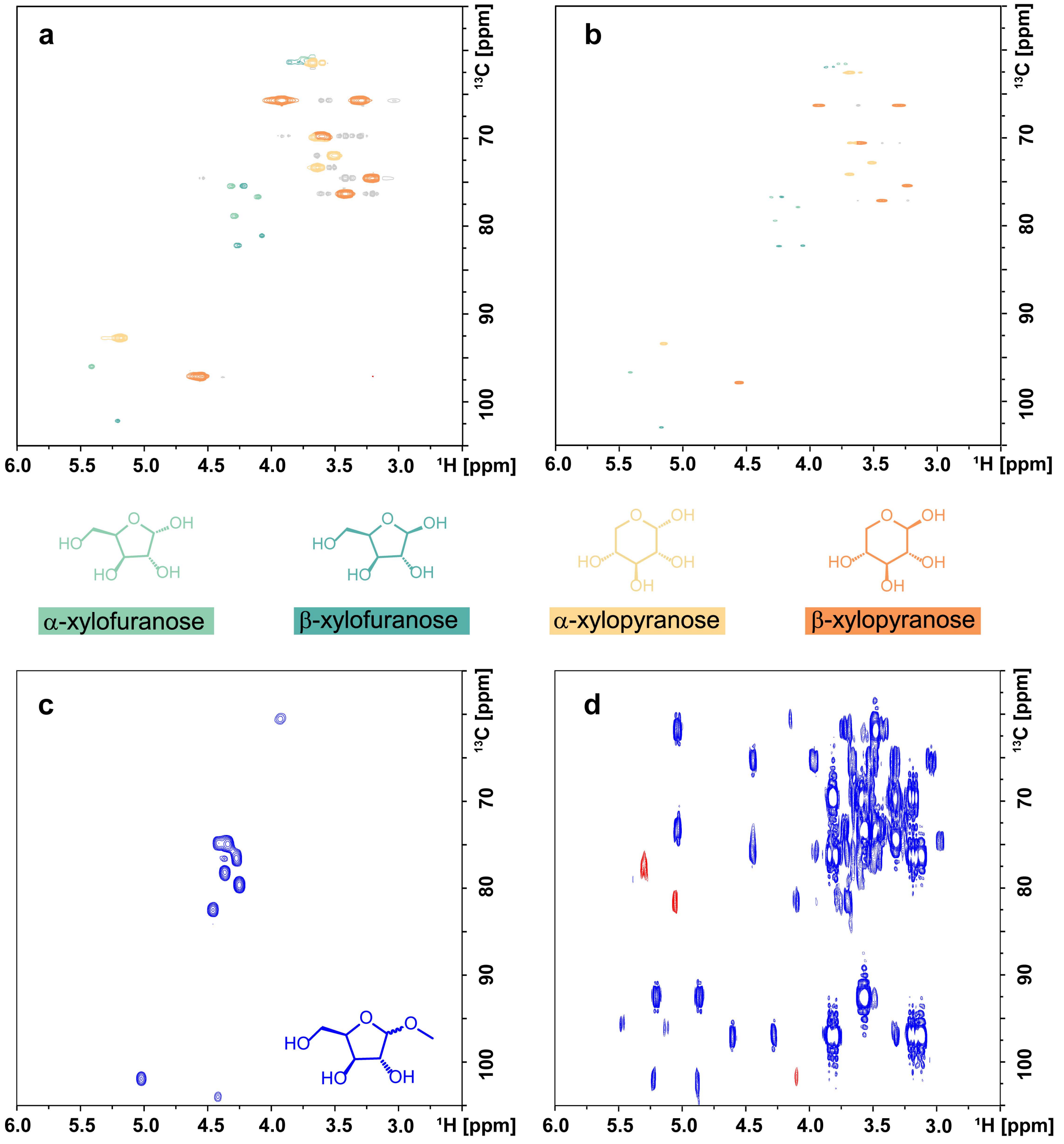

Figure S14. ^{1}H-^{13}C HSQC peak assignment for D-xylose tautomers at (a) 25°C and (b) 140°C. The sample was dissolved in D_2O due to high solubility. Trace of acetonitrile was added as the reference. (c) The ^{1}H-^{13}C HSQC of methyl-d-xylofuranoside, and (d) the ^{1}H-^{13}C HMBC spectrum of D-xylose

tautomers at 140°C in D_2O. The cross peaks linking the C1 peaks to the C2-C4 peaks are highlighted in red.

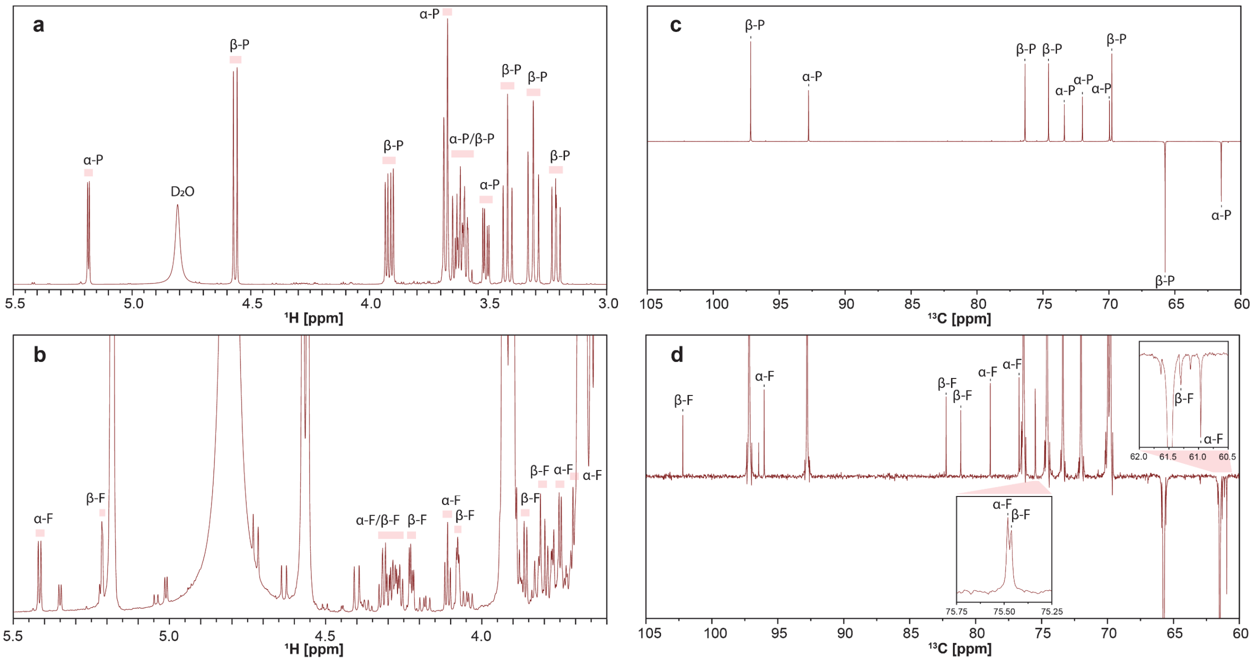

Figure S15. (a)-(b) ^{1}H and (c)-(d) ^{13}C MR peak assignment for D-xylose tautomers at 25°C in D_2O. Chemicals shifts of pyranose are labelled in (a) and (c), while panel (b) and (d) zoom in to show the furanose peaks. α/β-P denote α/β-xylopyranose peaks and α/β-F denote α/β-xylofuranose peaks.

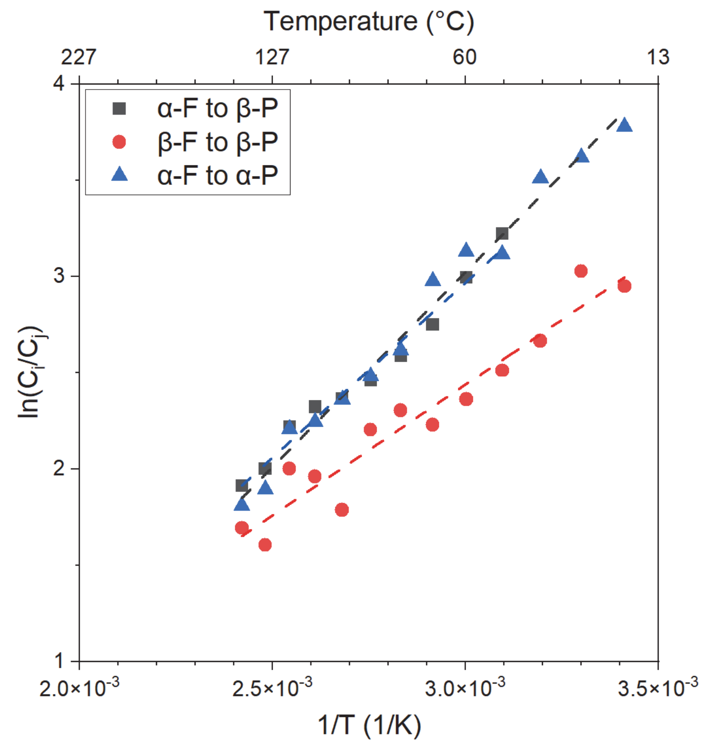

Figure S16. Linear regression of equilibrium constant for xylose isomerization reactions in 1,4-dioxane, with C_i/C_j being the tautomer concentration ratios and $1/T$ being the inverse temperature.

Table S3. Summary of linear regression data and the calculated thermodynamic parameters of xylose tautomerization in 1,4-dioxane.

	**α-F to β-P**	**β-F to β-P**	**α-F to α-P**
**Slope (K)**	1820.8	1356.6	2027.3
**Error in slope (K)**	91.8	113.6	75.9
**y-intercept (-)**	-2.49	-1.63	-3.06
**Error in y-intercept (-)**	0.25	0.33	0.22
**R^2**	0.980	0.928	0.985
**$\Delta H°_{app}$ (kJ/mol)**	-15.1 ± 0.8	-11.3 ± 0.9	-16.8 ± 0.6
**$\Delta S°_{app}$ (J/mol·K)**	-20.7 ± 2.1	-13.6 ±2.7	-25.4 ± 1.8

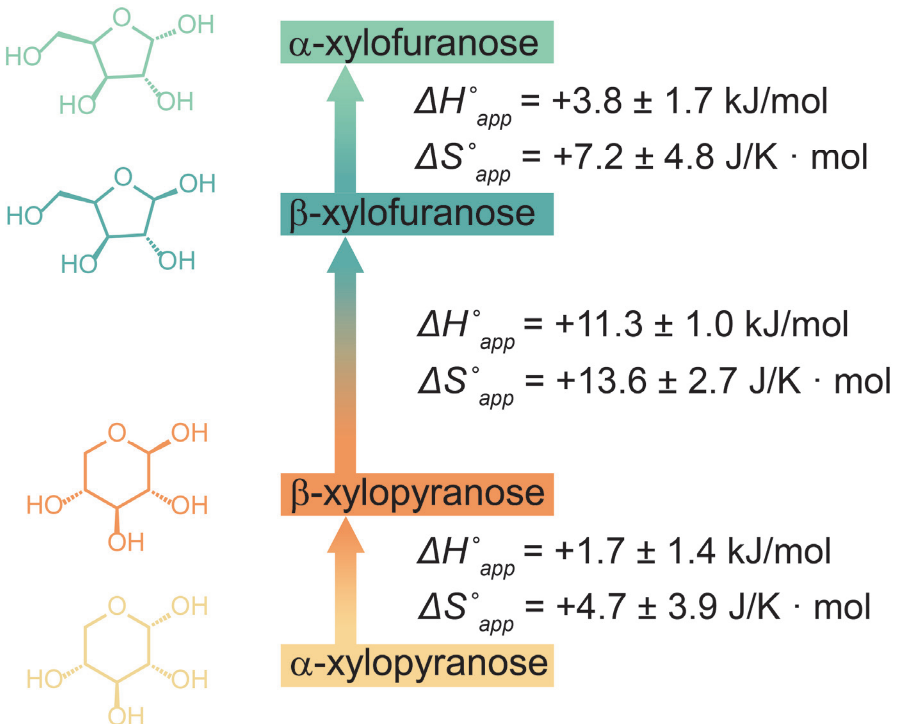

Figure S17. The summary of standard changes in enthalpy and entropy in xylose tautomerization in 1,4-dioxane.

S8. *Ab initio* calculations

Table S4. Electronic state properties of respective aldehydes calculated using M062X/def2-TZVP level of theory. Notations: formaldehyde (FA), propanal (PA), dodecanal (DA), benzaldehyde (BnA), fluorobenzaldehyde (BnAF), highest occupied molecular orbital energy (HOMO), lowest occupied molecular orbital energy (LUMO), electronegativity (μ), chemical hardness (η), and electrophilicity index of the molecule (EI), and electrophilicity index of the carbonyl carbon ($EI_{\text{-CHO}}$).

	FA	PA	DA	BnA	BnAF
**HOMO (Ha)**	-0.35	-0.33	-0.33	-0.32	-0.32
**LUMO (Ha)**	0.00	0.02	0.02	-0.04	-0.04
**μ (Ha)**	0.18	0.16	0.15	0.18	0.18
**η (Ha)**	0.17	0.17	0.17	0.14	0.14
**EI (Ha)**	0.09	0.07	0.07	0.11	0.12
**EI (kcal/mol)**	55.81	45.10	43.61	70.86	76.01
**$EI_{\text{-CHO}}$ (kcal/mol)**	42.67	29.19	28.07	14.24	14.71
**Spin density of C (radical)**	0.76	0.65	0.64	0.20	0.19
**Charge on C (neutral)**	0.05	0.12	0.19	0.09	0.10
**Charge on C (radical)**	-0.42	-0.27	-0.30	-0.08	-0.07
**Fukui Index of C**	-0.47	-0.39	-0.49	-0.16	-0.17

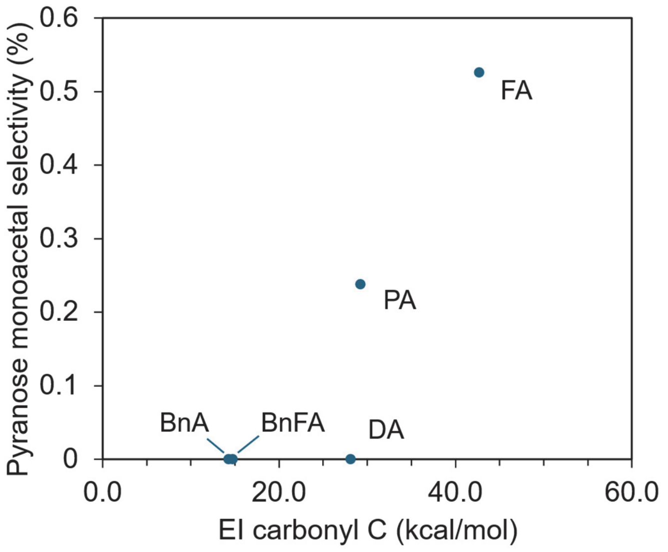

Figure S18. Correlation between the equilibrium pyranose monoacetal selectivity measured in the operando HSQC experiments and the electrophilicity index of the carbonyl carbon in different aldehydes used in Figure 3. The pyranose monoacetal selectivity is defined as $\text{Selectivity (mol\%)} = \frac{\text{Monoacetal}_{\text{pyranose}}}{\text{Monoacetal}_{\text{pyranose}}+\text{Monoacetal}_{\text{furanose}}} \times 100\%$.

Table S5. Zero-point energies (ZPE), entropies (S), and formation free energies (G) calculated by DFT for reaction intermediates involved in acidic formylation of α-D-xylopyranose (*alpha* xylopyranose) to 5-O-diformylene-D-xylofuranose (DFX) at 333.15 K. Formation free energies are referenced to isolated HCHO, H_2O, H_3O^+, and α-D-xylopyranose.

Species	ZPE (eV)	S(J/(mol*K))	G (eV)
**HCHO**	0.74	222.37	0.00
**H_2O**	0.59	198.06	0.00
**H_3O^+**	0.94	206.86	0.00
**$HCHOH^+$**	1.01	230.36	0.65
***alpha* xylofuranose**	4.52	429.76	0.00
***beta* xylofuranose**	4.52	435.75	0.08
***alpha* xylopyranose**	4.55	409.35	-0.10
***beta* xylopyranose**	4.53	414.95	0.01
***alpha* Int1**	4.75	412.51	-0.17
***beta* Int1**	4.72	426.93	0.02
***alpha* Int2**	4.74	416.66	-0.05
***beta* Int2**	4.72	423.36	0.07
***alpha* Int3**	4.74	418.50	-0.37
***beta* Int3**	4.74	415.14	0.07
***alpha* Int4**	4.72	429.62	-0.25
***beta* Int4**	4.69	443.06	0.95
***alpha* Int5**	4.76	409.40	-0.32
***beta* Int5**	4.76	412.52	-0.19
***alpha* DFX**	4.95	414.43	-0.67
**1a**	5.79	485.71	-1.37
**1b**	5.80	479.36	-1.57
**2a**	5.77	452.95	-2.12
**2b**	5.79	483.70	-1.48
**3a**	5.82	472.58	-1.81
**3b**	5.78	485.40	-1.66
**4a**	5.82	463.23	-1.31
**5a**	5.75	455.82	-2.03
**5b**	5.73	464.73	-2.02
**6a**	5.79	475.26	-1.56
**6b**	5.80	469.81	-1.61
**7a**	5.75	460.83	-1.80
**7b**	5.77	476.76	-1.11
**8a**	5.74	498.90	-1.24
**9a**	5.78	479.44	-1.56
**9b**	5.79	481.70	-1.41
**10a**	5.75	459.71	-1.82

**10b**	5.78	489.42	-1.26
**11a**	5.77	500.63	-1.32
**11b**	5.77	494.34	-1.43
**12b**	5.76	457.56	-2.06
**13a**	5.79	469.68	-1.58
**13b**	5.76	478.45	-1.70
**14b**	5.74	459.34	-1.60
**15b**	5.78	486.20	-0.46
**16a**	5.78	488.65	-1.31
**16b**	5.80	483.67	-1.41
**17a**	5.75	473.55	-1.72
**18a**	5.75	469.73	-1.82
**19a**	5.77	501.74	-1.67
**19b**	5.76	478.26	-1.86
**20b**	5.78	483.53	-0.73
**M1a**	5.09	411.55	-1.31
**M1b**	5.07	416.11	-1.21
**M2a**	5.08	413.54	-1.13
**M3a**	5.07	417.10	-1.09
**M3b**	5.05	420.82	-1.04
**M4a**	5.07	416.45	-1.16
**M4b**	5.05	420.80	-1.00
**M5a**	5.06	415.93	-0.99
**M5b**	5.05	421.04	-0.90
**M6a**	5.06	419.16	-1.33
**M6b**	5.06	418.01	-0.97
**M7a**	5.09	420.26	-1.40
**M7b**	5.07	420.66	-1.23
**M8a**	5.10	415.43	-1.31
**M8b**	5.09	417.76	-1.29
**M11a**	5.03	434.53	-1.31
**M11b**	5.01	445.90	-0.10
**M12a**	5.03	433.25	-0.98
**M12b**	5.03	433.25	-0.98
**21a**	5.99	480.08	-1.66
**21b**	6.02	465.23	-1.54
**23a**	6.00	484.02	-1.68
**23b**	6.00	483.24	-1.37
**24a**	5.98	466.01	-1.83
**24b**	5.98	456.19	-1.73
**27a**	5.97	493.21	-1.31

**27b**	5.95	502.09	0.09
**25a**	5.99	494.08	-1.98
**25b**	6.01	471.14	-0.87
**26a**	5.97	494.10	-1.12
**26b**	5.96	490.55	0.26
**28a**	5.96	464.63	-2.10
**M15a**	5.29	421.22	-1.42
**M15b**	5.26	425.73	-0.40
**M16a**	5.29	419.12	-1.36
**M16b**	5.29	419.29	-0.36
**M14a**	5.27	416.66	-1.21
**M14b**	5.27	416.28	-0.33
***alpha* RO3**	5.06	437.47	-1.27
***beta* RO3**	5.06	421.54	-1.03
**M17a**	5.04	434.40	-1.84
**M17b**	5.06	422.65	-0.57

Table S6. Solvation energies ($E_{solvent} - E_{gas}$) and free energies ($G_{solvent} - G_{gas}$) for key reaction intermediates involved in acidic formylation of α-D-xylopyranose (*alpha* xylopyranose) to 5-O-diformylene-D-xylofuranose (DFX) in 1,4-dioxane at 333.15 K.

**Species**	**$E_{solvent} - E_{gas}$ (eV)**	**$G_{solvent} - G_{gas}$ (eV)**
**formaldehyde**	-0.07	-0.07
**water**	-0.14	-0.14
***alpha* xylofuranose**	-0.44	-0.45
***alpha* xylopyranose**	-0.44	-0.47
***alpha* Int 1**	-0.42	-0.44
***alpha* Int 2**	-0.43	-0.46
***alpha* Int 3**	-0.41	-0.41
***beta* Int 3**	-0.46	-0.47
***alpha* Int 4**	-0.45	-0.46
***alpha* Int 5**	-0.43	-0.45

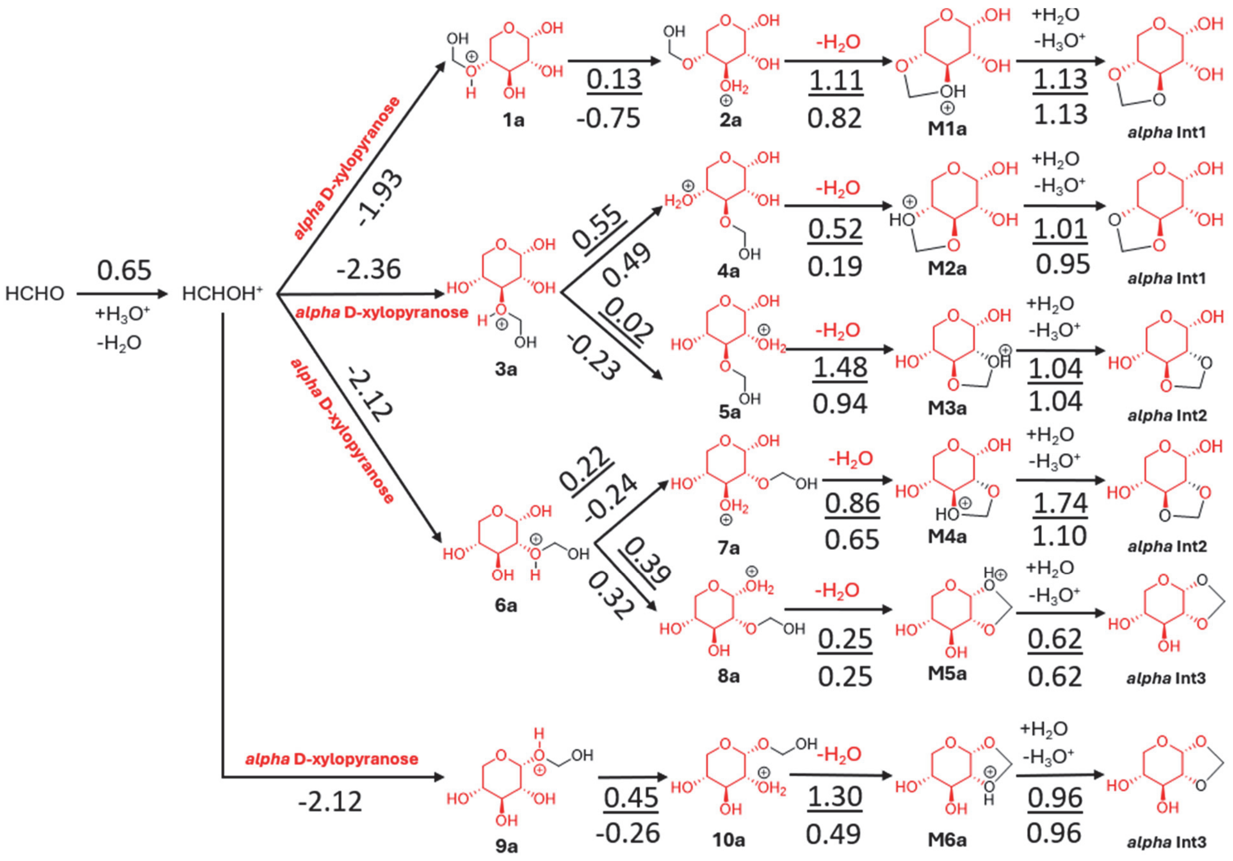

Figure S19. Elementary steps that were considered and the associated calculated reaction free energies (non-underlined, ΔG (eV)) and activation free energies (underlined, G_a (eV)) for α-D-xylopyranose (*alpha* xylopyranose) formylation to Int 1α (*alpha* Int1), Int 2α (*alpha* Int2), and Int 3α (*alpha* Int3).

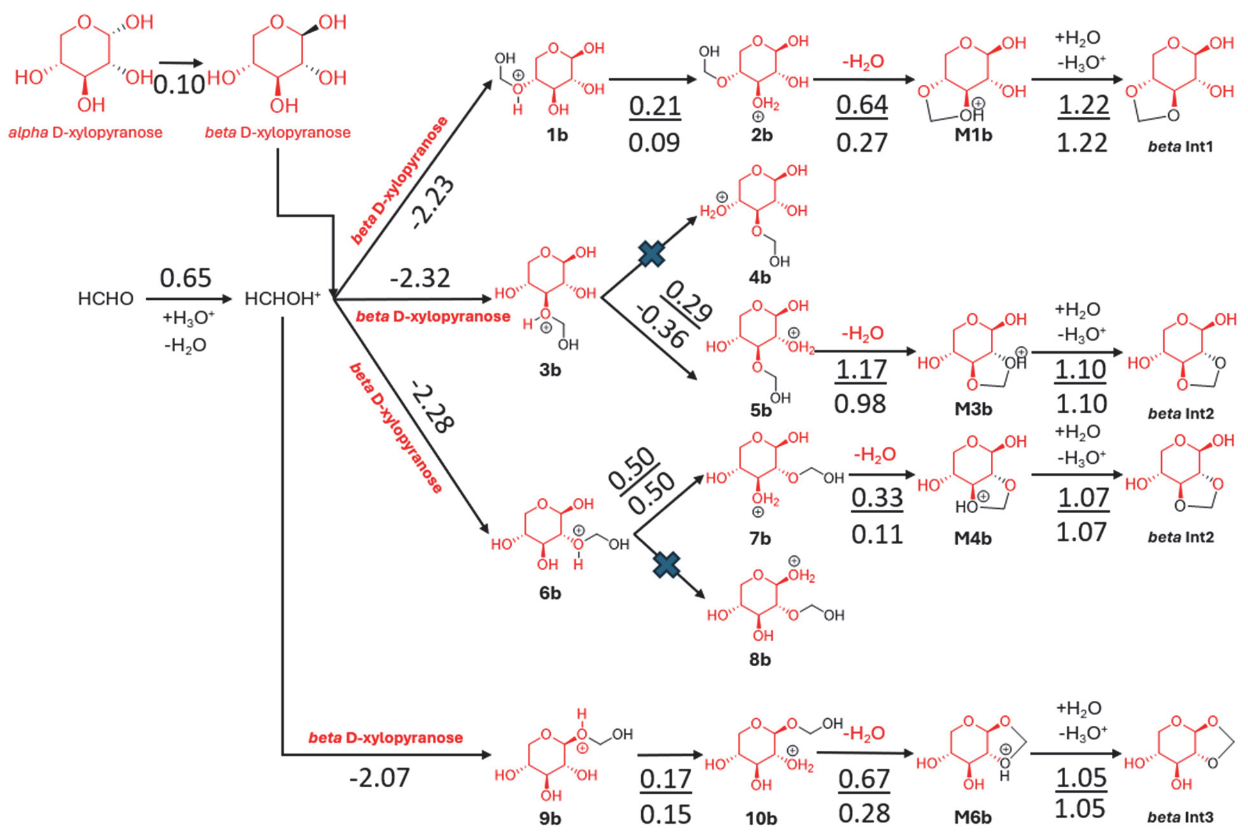

Figure S20. Elementary steps that were considered and the associated calculated reaction free energies (non-underlined, ΔG (eV)) and activation free energies (underlined, G_a (eV)) for β-D-xylopyranose (*beta* xylopyranose) formylation to Int 1β (*beta* Int1), Int 2β (*beta* Int2), and Int 3β (*beta* Int3). The cross sign indicates that a stable structure of the product involved in the corresponding elementary step was not found.

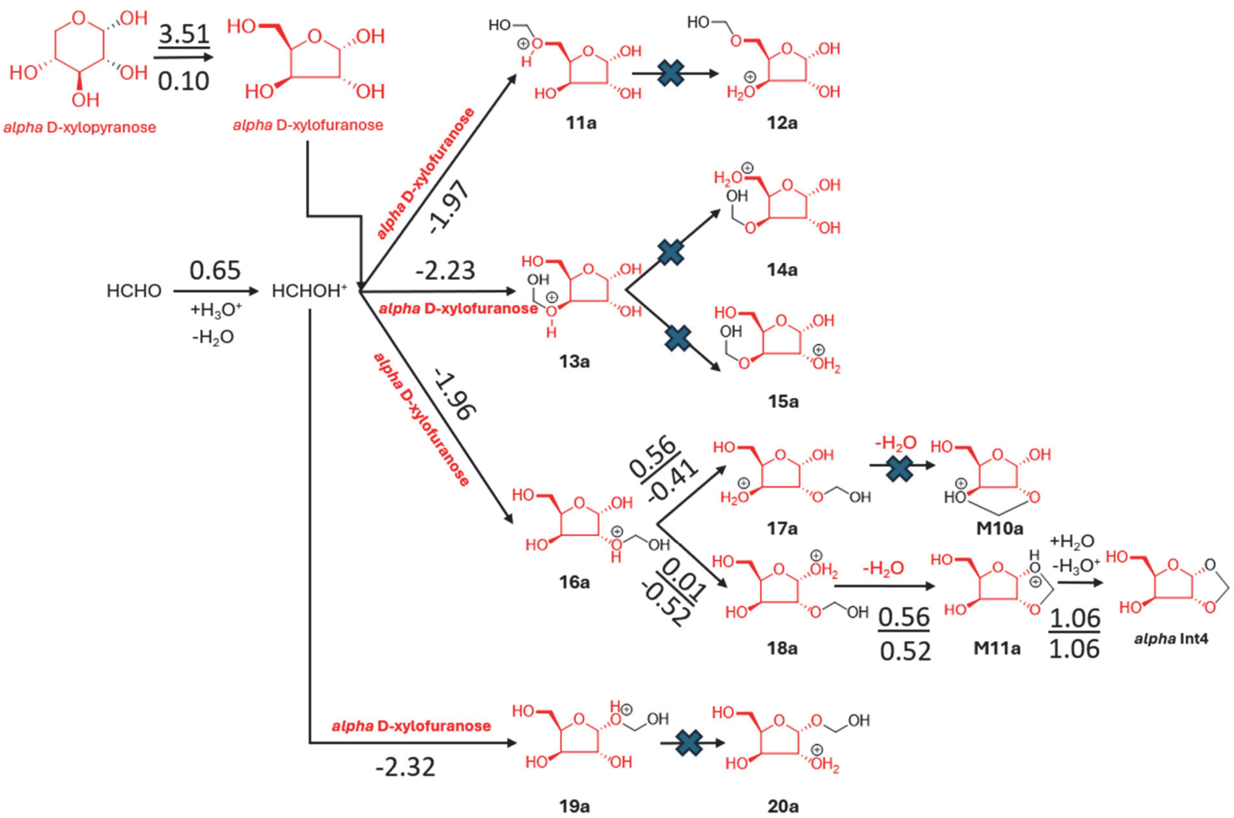

Figure S21. Elementary steps that were considered and the associated calculated reaction free energies (non-underlined, ΔG (eV)) and activation free energies (underlined, G_a (eV)) for α-D-xylofuranose (*alpha* xylofuranose) formylation to Int 4α (*alpha* Int4) and Int 5α (*alpha* Int5). The cross sign indicates that a stable structure of the product involved in the corresponding elementary step was not found. The barrier of the tautomerization step was derived in the presence of one explicit water molecule.

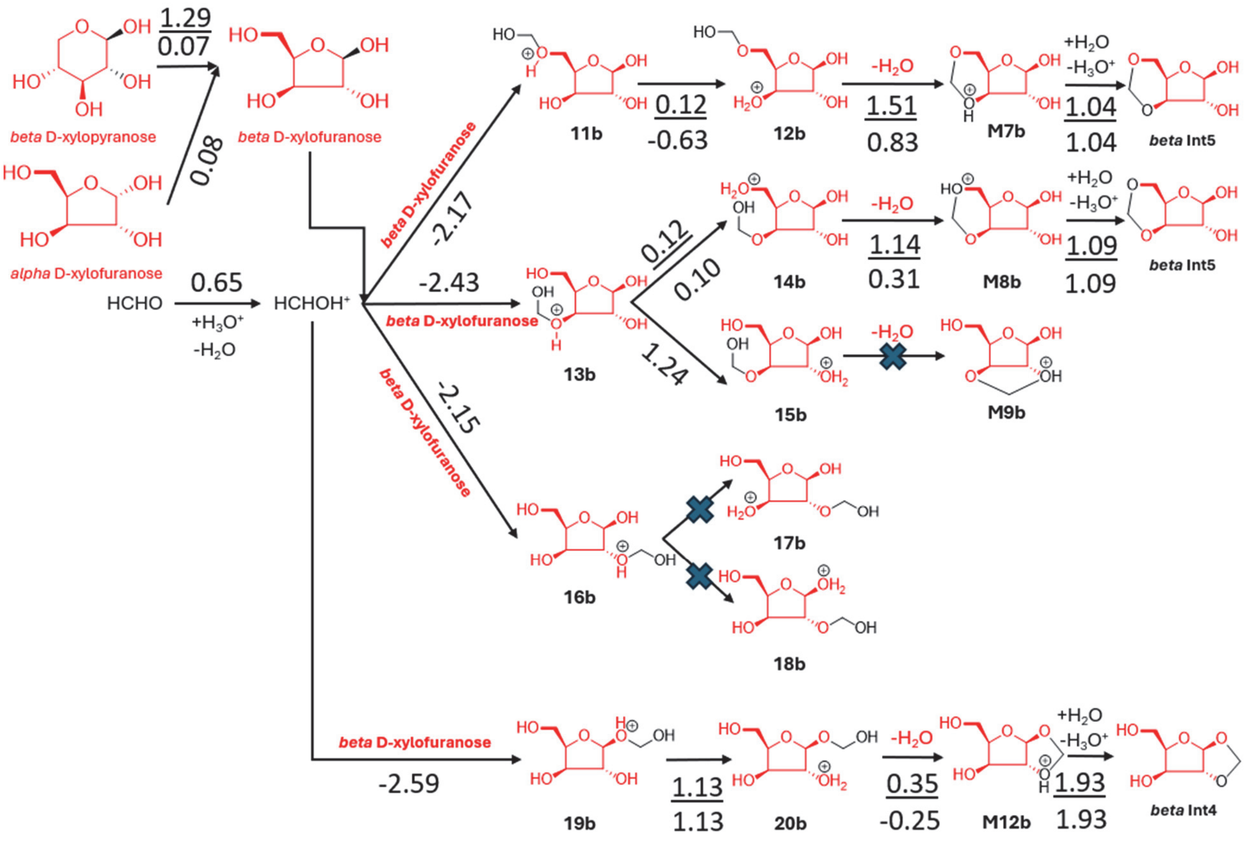

Figure S22. Elementary steps that were considered and the associated calculated reaction free energies (non-underlined, ΔG (eV)) and activation free energies (underlined, G_a (eV)) for β-D-xylofuranose (*beta* xylofuranose) formylation to Int 4β (*beta* Int4) and Int 5β (*beta* Int5). The cross sign indicates that a stable structure of the product involved in the corresponding elementary step was not found. The barrier of the tautomerization step was derived in the presence of one explicit water molecule.

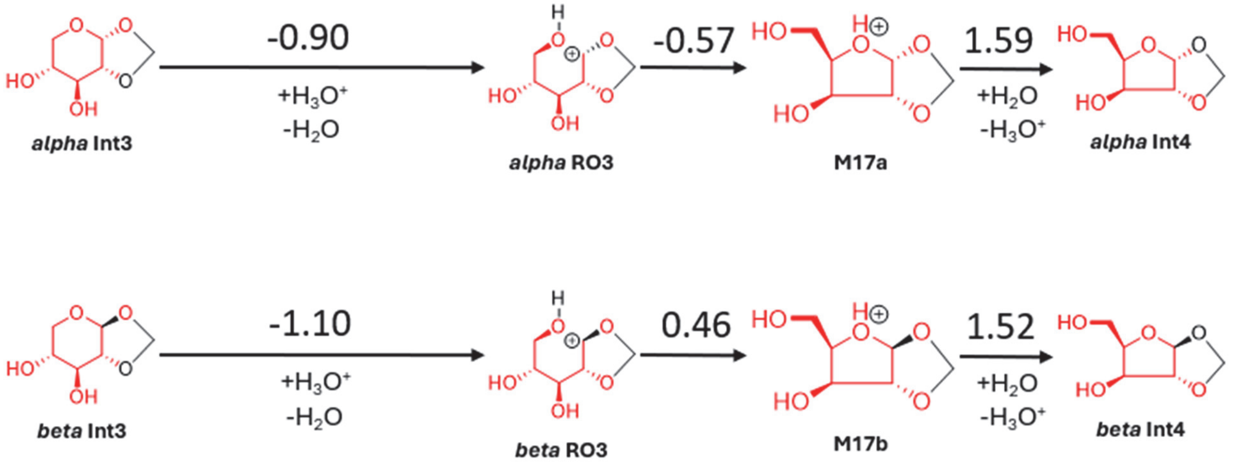

Figure S23. Elementary steps that were considered and the associated calculated reaction free energies (ΔG (eV)) for the conversion of Int 3 to Int 4.

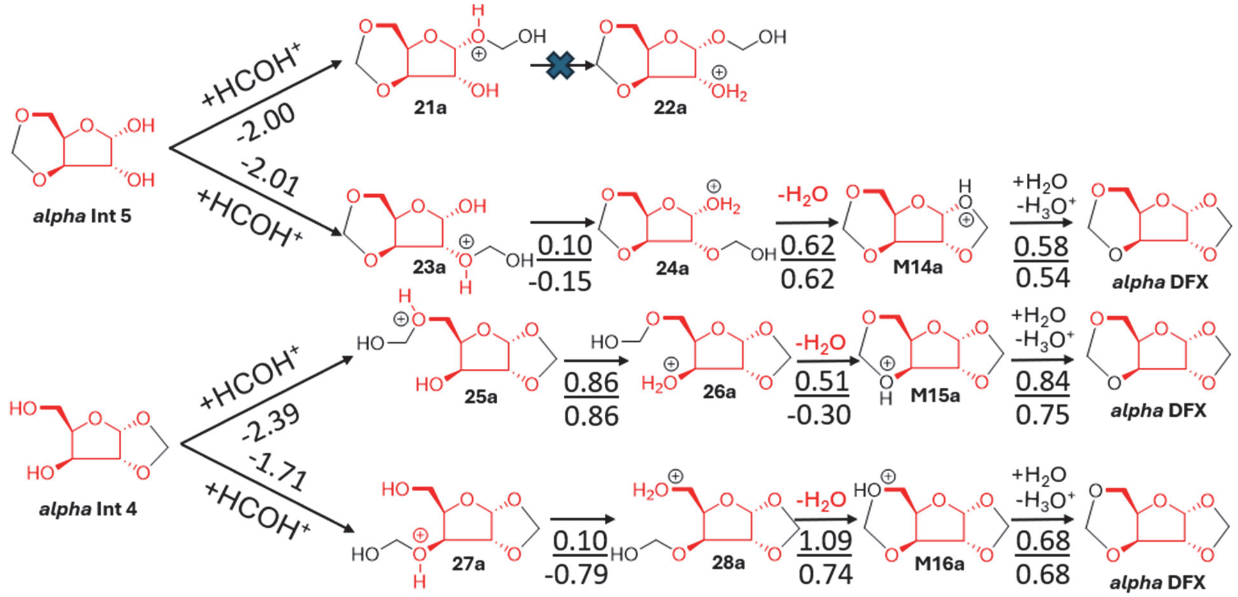

Figure S24. Elementary steps that were considered and the associated calculated reaction free energies (non-underlined, ΔG (eV)) and activation free energies (underlined, G_a (eV)) for Int 4α (*alpha* Int4) and Int 5α (*alpha* Int5) formylation to DFXα (*alpha* DFX). The cross sign indicates that a stable structure of the product involved in the corresponding elementary step was not found.

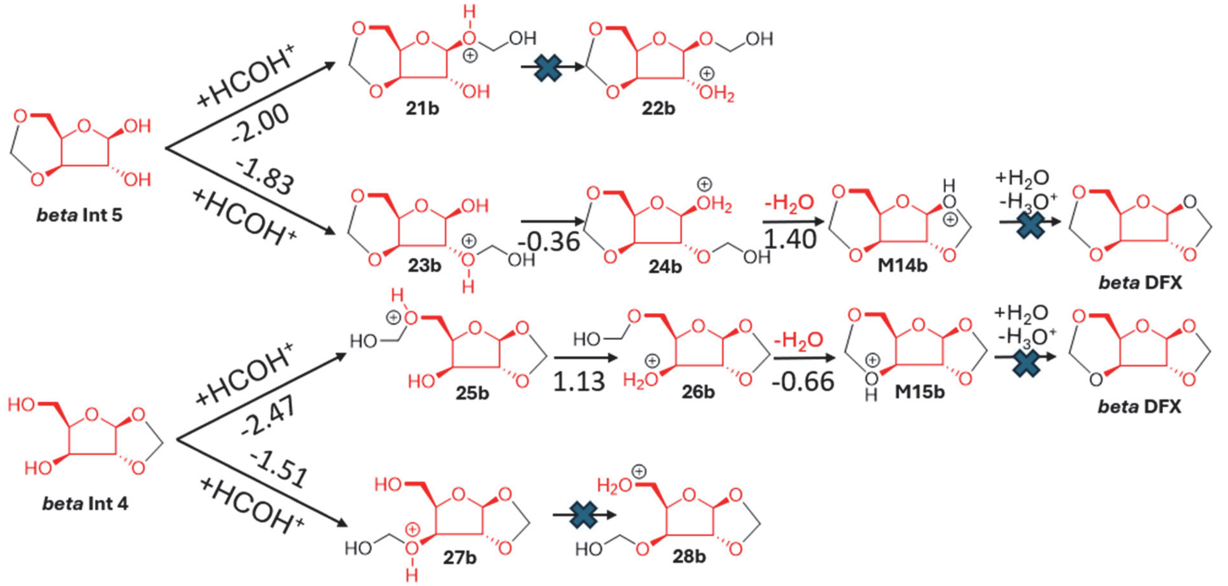

Figure S25. Elementary steps that were considered and the associated calculated reaction free energies (non-underlined, ΔG (eV)) and activation free energies (underlined, G_a (eV)) for Int 4β (*beta* Int4) and Int 5β (*beta* Int5) formylation to DFXβ (*beta* DFX). The cross sign indicates that a stable structure of the product involved in the corresponding elementary step was not found.

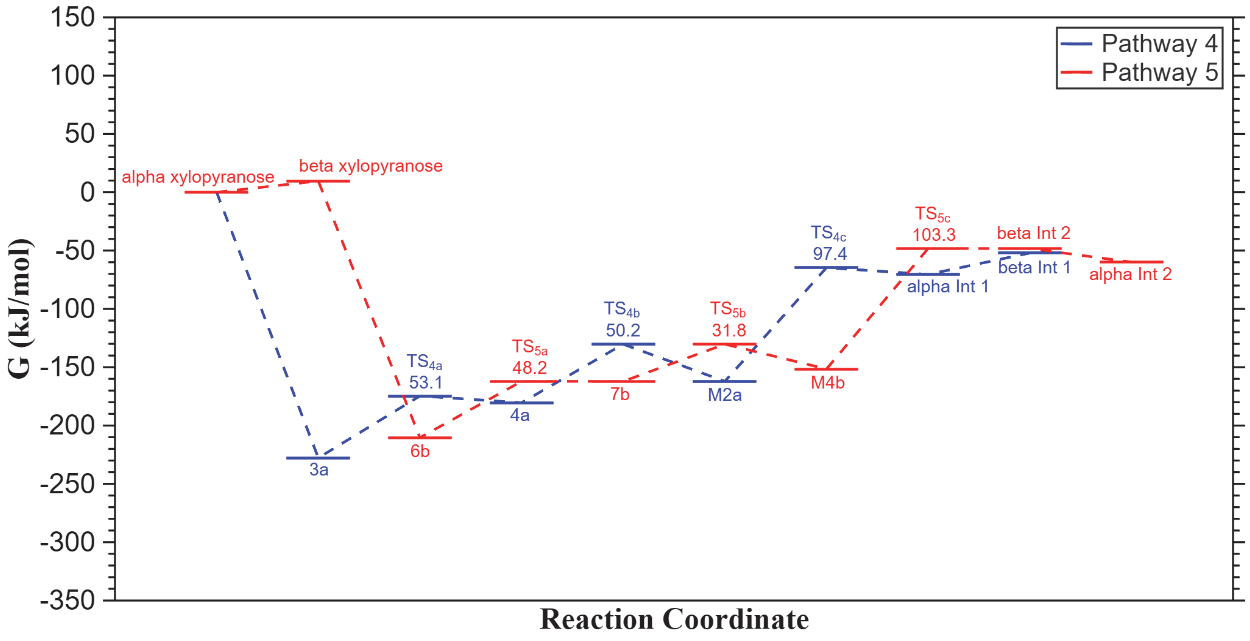

Figure S26. Relative free energies (G, kJ/mol) calculated by DFT-calculated for key reaction intermediates involved in the lowest barriers pathways for conversion of *alpha* xylofuranose to Int 1 and Int 2 at 60 °C. Numbers denote the activation free energies (G_a, kJ/mol) relative to initial states from the left of the respective elementary steps. Notations are consistent with Figure S19-S25.

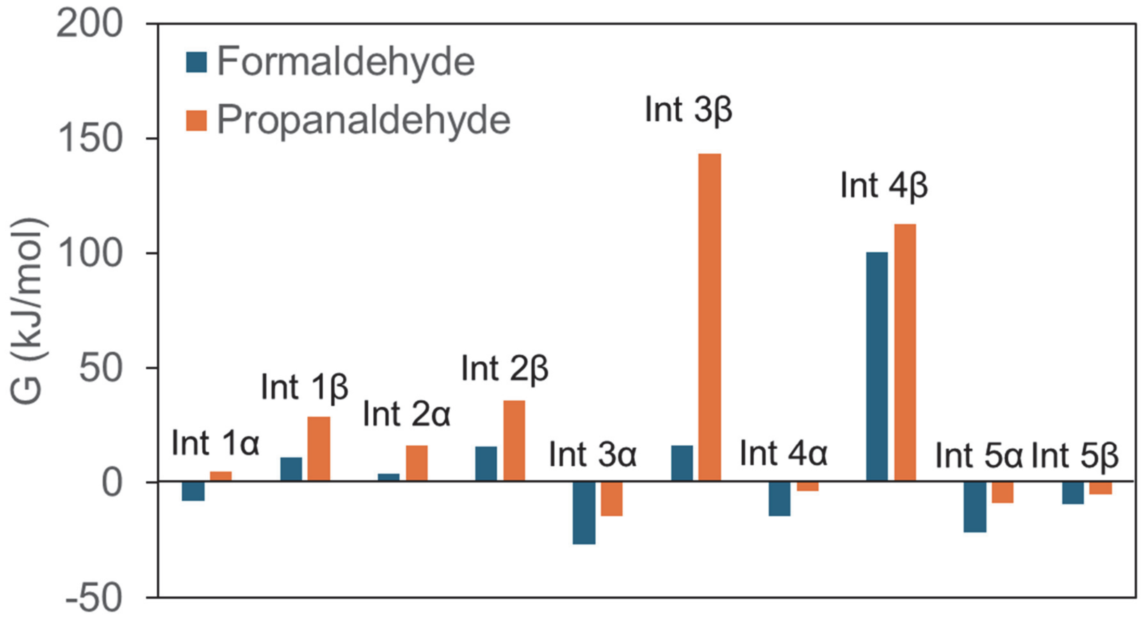

Figure S27. DFT-calculated formation free energies (G, kJ/mol) of key reaction intermediates involved in the conversion of *alpha* xylofuranose to Int 1, Int 2, Int 3, Int 4, and Int 5 at 60 °C.

S9. Acetalization of other monosaccharides

To access the applicability of the proposed reaction mechanism to other monosaccharides, we reacted other pentoses, including arabinose and lyxose, with formaldehyde and dodecanal. The unique hydroxyl configurations in these different sugar types could be used to explore the role of hydroxyl orientations and the resulting cyclic acetal ring strain on the product distribution. The comparison between formaldehyde and dodecanal further allowed us to expand the role of carbonyl electrophilicity. However, instead of tracking the evolution of individual acetals formed from different sugars, we focused on comparing the sum of monoacetals and the sum of diacetals among the acetalization products. This simplification allowed us to highlight the key mechanistic influencers without the tedious isolation and identification of many different intermediates, since the NMR chemical shifts of the acetals of different sugars significantly deviated from the values of those of xylose. Conventional GC-MS-FID allowed differentiation of mono- and diacetals based on mass fragments without the need to fully elucidate the isomer structures. Following our previous work, product quantification was performed using effective carbon numbers with n-decane as the internal standard.[13] The results are summarized in Figure S28.

Compared to xylose, both arabinose and lyxose formed significantly less diacetals with either aldehyde. This is expected to be a result of the hydroxyl configurations unique to each sugar. Compared to xylose, although the C1-C2 diol of arabinofuranose can be cis-positioned (the β-anomer), the C3-C4 diol of arabinofuranose is always in the trans configuration, in which case acetalization of both pairs of diols to form a furanose diacetal is energetically unfavoured. In contrast, β-arabinopyranose was much more likely to form diacetals due to the cis-positioned diol pairs. The slightly higher yield of arabinose-diacetal when reacted with formaldehyde than with

dodecanal might stem from the higher electrophilicity of FA, favouring pyranose acetalization over DA.

A similar trend of the product distribution was observed in lyxose acetalization, but the reasoning was different. Lyxose has no cis-positioned vicinal hydroxyls in the pyranose form, whereas both pairs of diols of β-lyxofuranose are cis-positioned, potentially favouring furanose diacetals during acetalization. However, both pairs of diols are oriented in the same direction, rather than opposite directions as in the case of xylofuranose, which might impose an additional steric hindrance when both diol pairs were acetalized. This may explain why lyxose formed significantly less diacetal than xylose did when reacting with both FA and DA. During the reaction with DA, lyxose formed no detectable diacetals, likely due to the much longer alkyl chain of DA causing a prohibitively high steric hindrance against any formation of diacetals.

Overall, this preliminary expansion of sugar substrates suggested that the unique hydroxyl configuration of each sugar, together with the electrophilicity of the aldehyde used during the reaction significantly affected the distribution of mono- and diacetals. To fully elucidate the acetalization pathways of each sugar type would require the identification and tracking of each acetal formed during the reaction and will be considered in future work.

.

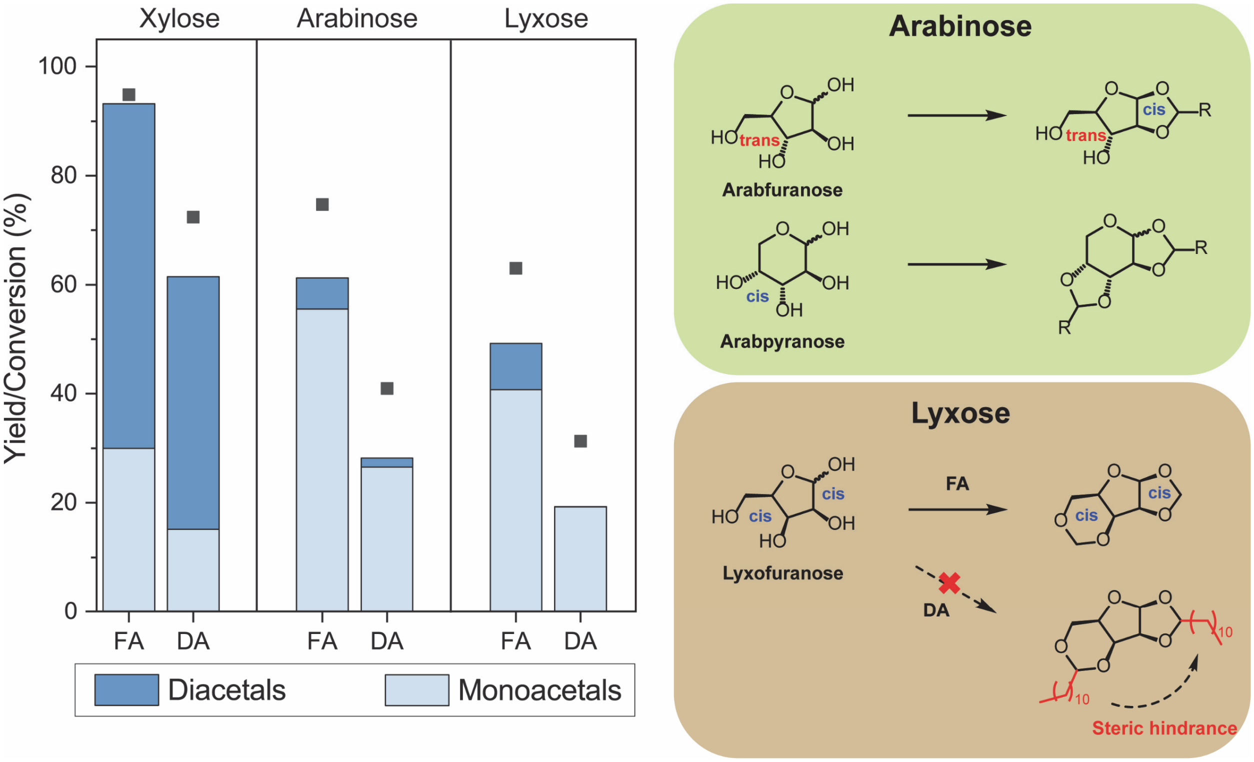

Figure S28. The mono- and diacetal product distribution of various monosaccharides' acetalization with formaldehyde (FA) and dodecanal (DA). Reactions were conducted at 60°C for 4 h in dioxane with a 2:1 aldehyde to xylose molar ratio and 1.6 vol.% H_2SO_4. Product yields were quantified using GC-FID with effective carbon numbers as per our previous work.[13] The proposed reaction pathways are also included.

S10. Validation of Mechanistic Applicability under Practical Acetalization Conditions

To verify if the mechanistic insights derived from operando NMR experiments in 1,4-dioxane-d8 with 1.6% D_2SO_4 remain valid under more practical catalytic conditions, we performed additional xylose acetalization experiments with formaldehyde using a HY-type zeolite as a heterogeneous Brønsted acid catalyst in 2-methyltetrahydrofuran (Me-THF), a greener alternative solvent to dioxane. The reactions were conducted under otherwise comparable conditions, and the concentrations of various monoacetals and DFX were quantified over a 6 h reaction period by HPLC, since Me-THF does not have a commercially available deuterated analogue for NMR measurements (Figure S29).

The overall carbon balance was slightly lower than in the deuterated homogeneous system, which can be attributed to two factors: (1) the faster proton transfer kinetics compared to D_2SO_4 likely accelerated xylose dehydration, and (2) the presence of a small number of Lewis acid sites in HY zeolite may have promoted side reactions such as xylose isomerization (see Table S7), producing minor unquantified side products like xylulose and its derivatives.[13]

Nevertheless, the temporal evolution of monoacetal and diacetal species in this system closely mirrored what was observed in the operando HSQC NMR experiments with dioxane and the homogeneous acid (Figure 3a). This consistency confirms that the acetalization mechanism derived from the model system—particularly the sequence of pyranose/furanose interconversion and acetalization steps—remains applicable under realistic catalytic conditions using a green solvent and a heterogeneous Brønsted acid catalyst.

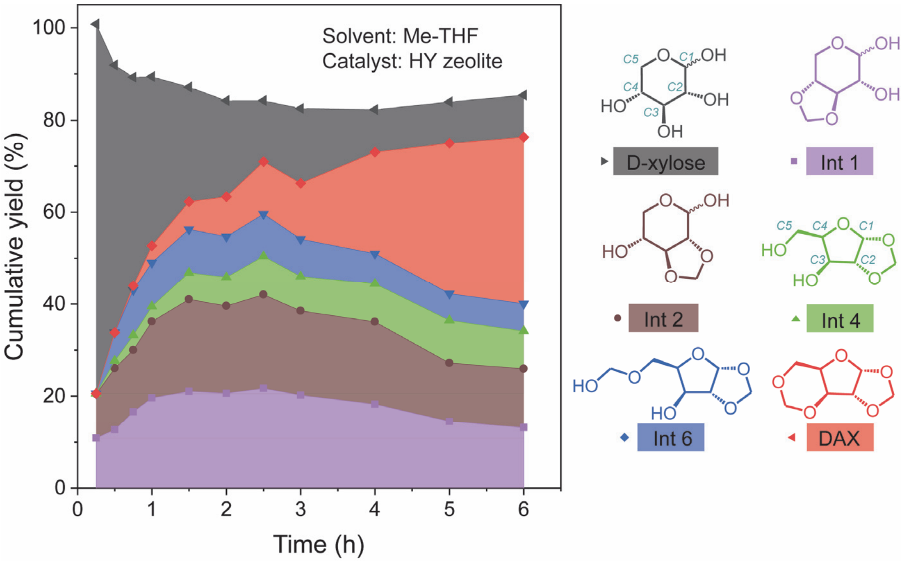

Figure S29. The molar fraction of xylose, intermediates, and products as a function of reaction time when reacting it with formaldehyde as measured by HPLC. The reaction was conducted with HY zeolite as a heterogeneous Brønsted catalyst and in 2-methylTHF as a greener alternative to 1,4-dioxane (80°C with a 2:1 formaldehyde to xylose molar ratio. HY zeolite (SiO_2:Al_2O_3 = 80:1) was loaded at 80 g/L solvent). Full characterization of HY zeolite can be found in prior work and summarized in Table S7.[13]

Table S7. Characterization results of the HY zeolite used in this experiment.[13]

**Topology**		**FAU**
**Maximum diameter of a sphere that can diffuse along: (Å)**	**a**	7.35
	**b**	7.35
	**c**	7.35
**Ring size (MR)**		12
**SiO_2/Al_2O_3 ratio**		80
**Channel dimensions (Å)**		7.4×7.4
**Total S_{BET} (m^2/g)**		784.3 ± 15.7
**Micropore area (m^2/g)**		558.3
**Total Pore volume (cm^3/g) @0.95 P/P_0**		0.58
**Micropore volume (cm^3/g)**		0.29
**Pore diameter (Å)**		7.78
**Crystallite size (Å)**		458.7
**LAS density (µmol/g)**		80.2
**BAS density (µmol/g)**		33.9

”

Reference

1. Bourmaud, C. L., Sun, S., Bornet, A. & Luterbacher, J. S. Operando monitoring of delignification processes using 2D 1H-13C HSQC NMR. *ACS Sustain. Chem. Eng.* In revision (2025).

2. Amiri, M. T., Bertella, S., Questell-Santiago, Y. M. & Luterbacher, J. S. Establishing lignin structure-upgradeability relationships using quantitative 1H-13C heteronuclear single quantum coherence nuclear magnetic resonance (HSQC-NMR) spectroscopy. *Chem. Sci.* **10**, 8135–8142 (2019).

3. Hu, K., Westler, W. M. & Markley, J. L. Simultaneous quantification and identification of individual chemicals in metabolite mixtures by two-dimensional extrapolated time-zero 1H-13C HSQC (HSQC0). *J. Am. Chem. Soc.* **133**, 1662–1665 (2011).

4. Massiot, D. *et al.* Modelling one- and two-dimensional solid-state NMR spectra. *Magn. Reson. Chem.* **40**, 70–76 (2002).

5. Bock, K. & Thøgersen, H. Nuclear Magnetic Resonance Spectroscopy in the Study of Mono- and Oligosaccharides. *Annu. Reports NMR Spectrosc.* **13**, 1–57 (1983).

6. Hu, K., Ellinger, J. J., Chylla, R. A. & Markley, J. L. Measurement of absolute concentrations of individual compounds in metabolite mixtures by gradient-selective time-zero 1H- 13C HSQC with two concentration references and fast maximum likelihood reconstruction analysis. *Anal. Chem.* **83**, 9352–9360 (2011).

7. Tsukiashi, A. *et al.* Application of spin-crossover water soluble nanoparticles for use as MRI contrast agents. *Sci. Rep.* **8**, 6–11 (2018).

8. Grashei, M., Hundshammer, C., van Heijster, F. H. A., Topping, G. J. & Schilling, F. PH dependence of T2 for hyperpolarizable 13C-labelled small molecules enables spatially resolved pH measurement by magnetic resonance imaging. *Pharmaceuticals* **14**, (2021).

9. Lankhorst, D., Schriever, J. & Leyte, J. C. Determination of the Rotational Correlation Time of Water by Proton NMR Relaxation in H 2 17 O and Some Related Results. *Berichte der Bunsengesellschaft für Phys. Chemie* **86**, 215–221 (1982).

10. Gómez-Gallego, M. & Sierra, M. A. Kinetic Isotope Effects in the Study of Organometallic Reaction Mechanisms. *Chem. Rev.* **111**, 4857–4963 (2011).

11. Alexandersson, E. & Nestor, G. Complete 1H and 13C NMR spectral assignment of D-glucofuranose. *Carbohydr. Res.* **511**, 108477 (2022).

12. Schmidt, R. K., Karplus, M. & Brady, J. W. The anomeric equilibrium in D-xylose: Free energy and the role of solvent structuring. *J. Am. Chem. Soc.* **118**, 541–546 (1996).

13. Sun, S. *et al.* Size-Selective Functionalization of Sugars and Polyols Using Zeolites for Renewable Surfactant Production. *Angew. Chemie Int. Ed.* In press (2025). doi:10.1002/anie.202511282
